# TFAP2A regulates SGPP2 transcription to promote lipid accumulation and activate the Wnt/β-catenin signaling pathway to promote malignant progression in lung adenocarcinoma

**DOI:** 10.1186/s12967-026-07949-x

**Published:** 2026-03-04

**Authors:** Qingqing Li, Jiangying Li, Cui Zhang, Yuxuan Deng, Bowen Yang, Li Ma, Yuxiang Liu, Chunliang Wang, Linlin Xu, Jinhong Mei

**Affiliations:** 1 Department of Pathology, The First Affiliated Hospital, Jiangxi Medical College, Nanchang University, 17 Yongwaizheng Street, Nanchang, 330006 P.R. China; 2 Institute of Molecular Pathology, Nanchang University, Nanchang, 330006 China; 3 Department of Neurosurgery, The First affiliated hospital, Jiangxi Medical College, Nanchang University, Nanchang, 330006 China

**Keywords:** SGPP2, TFAP2A, S1P, Lipid accumulation, Lung adenocarcinoma

## Abstract

**Background:**

Lung adenocarcinoma (LUAD) is the leading cause of cancer-related mortality worldwide, highlighting the urgent need for additional molecular biomarkers and therapeutic targets. Transcription factor AP-2α (TFAP2A) is highly expressed in LUAD and is associated with poor prognosis. Sphingosine-1-phosphate phosphatae 2 (SGPP2/SPP2) has been implicated in tumor progression in multiple cancer types; however, its functional role in LUAD cells and the underlying mechanisms remain unclear.

**Methods:**

Bioinformatics analysis was conducted to elucidate the expression patterns of SGPP2 and TFAP2A. Quantitative real-time polymerase chain reaction (qRT-PCR), western blotting (WB), and immunohistochemistry (IHC) were performed to measure mRNA and protein expression levels. Cellular proliferation and cell cycle progression were evaluated using the Cell Counting Kit-8 (CCK-8) assay, 5-ethynyl-2’-deoxyuridine (EdU) assay, colony formation assay, and flow cytometry. Migratory and invasive capabilities were evaluated using transwell and wound-healing assays. Lipid metabolism was assessed by measuring triglyceride (TG) and total cholesterol (TC) levels, using Oil Red O and Nile Red fluorescence staining. The regulatory relationship between TFAP2A and the SGPP2 promoter was confirmed using chromatin immunoprecipitation (ChIP) and dual-luciferase reporter assays. Protein-protein interactions were investigated using co-immunoprecipitation (CoIP) assay. The in vivo tumorigenic potential was examined using a xenograft model in nude mice.

**Results:**

SGPP2 and TFAP2A were upregulated in LUAD. High SGPP2 expression is closely associated with lymph node metastasis. Functional experiments demonstrated that SGPP2 promotes LUAD cell proliferation, migration, and epithelial-mesenchymal transition (EMT). Under physiological conditions, TFAP2A transcriptionally activates SGPP2 in LUAD cells, whereas SGPP2 reciprocally inhibits TFAP2A expression. Downstream pathway analysis revealed that SGPP2 overexpression downregulated SGPP1 expression, leading to increased sphingosine-1-phosphate (S1P) levels in the cells. This, in turn, promotes intracellular lipid accumulation and phosphorylation of glycogen synthase kinase 3β (GSK3β) at Ser9, thereby facilitating the nuclear translocation of β-catenin. Consequently, CyclinD1 expression is upregulated, ultimately driving LUAD progression.

**Conclusion:**

SGPP2 and TFAP2A are highly expressed in LUAD. SGPP2, which regulates S1P levels and is transactivated by TFAP2A, promotes lipid accumulation and activates the Wnt/β-catenin signaling pathway to facilitate the progression of lung adenocarcinoma.

**Supplementary Information:**

The online version contains supplementary material available at 10.1186/s12967-026-07949-x.

## Background

Lung cancer has the highest global incidence and mortality rate worldwide. In 2023, there will be approximately 2.37 million new cases and over 1.8 million deaths worldwide, with persistently elevated incidence and mortality rates. Lung adenocarcinoma (LUAD) accounts for 40%–50% of non-small cell lung cancer (NSCLC) cases. Owing to its insidious onset and rapid progression, most patients are diagnosed at an advanced stage, resulting in a 5-year survival rate of < 20% [1–3]. Despite advances in medical research and pharmaceutical technology, novel treatment options and only modest improvements in overall outcomes have led to suboptimal [4].

Sphingosine-1-phosphate phosphatase 2 (SGPP2/SPP2) is a transmembrane protein encoded by the *SGPP2* gene. As a homologous gene specifically encoding sphingosine-1-phosphate (S1P) phosphatase, it catalyzes the dephosphorylation of S1P [5]. Studies have shown that SGPP2 exerts proinflammatory effects during inflammatory responses [6–9]. Moreover, dysregulated SGPP2 expression has been observed in various cancers, including pancreatic, prostate, and hepatocellular carcinomas (HCC), suggesting its potential involvement in cancer cell proliferation, metastasis, and lipid metabolism [10–14].

Transcription factor AP-2α (TFAP2A), a core member of the AP-2 family, exhibits cancer type-specific functions. In pancreatic cancer, it activates *MYC* to promote liver metastasis [15], whereas in HCC, it suppresses lipogenic genes and acts as a tumor suppressor [16]. However, in LUAD, high TFAP2A expression is associated with tumor progression and poor prognosis. Studies have shown that TFAP2A transcriptionally activates *CTHRC1* expression, thereby promoting fatty acid metabolism. However, its regulatory network involving lipid metabolism and signaling pathways remains unclear [17, 18]. Emerging evidence indicates that lipid metabolic reprogramming provides energy and membrane structural components for tumor cells, remodels the tumor microenvironment (TME), and modulates tumor invasion and metastasis [19, 20]. Lipid metabolites, such as unsaturated fatty acids, can regulate the Wnt/β-catenin pathway by modulating glycogen synthase kinase 3β(GSK3β) activity [21, 22]. Nevertheless, the expression pattern of SGPP2 and its regulatory roles in lipid metabolism and signaling pathways in LUAD remain unclear. Given the transcriptional regulatory properties of TFAP2A and the metabolic regulatory function of SGPP2, we hypothesized that TFAP2A transcriptionally regulates SGPP2 expression, leading to activation of the Wnt/β-catenin pathway and aberrant lipid metabolism, ultimately promoting LUAD progression.

In this study, we explored the functional role and regulatory mechanism of SGPP2 in LUAD using bioinformatics database analysis, clinical sample validation, and cellular function experiments. Our findings delineate the critical role of the “TFAP2A-SGPP2- lipid metabolism -Wnt/β-catenin” regulatory axis in LUAD progression, thereby providing a theoretical basis for LUAD diagnosis and targeted therapy.

## Materials and methods

### Bioinformatics analysis

The gene expression datasets GSE75037, GSE116959, and GSE118370 were retrieved from the Gene Expression Omnibus (GEO) database. Following initial visualization, the datasets were subjected to a standardized quality assessment using the Zerocoding platform (https://zc.bioinfosci.cn/). Principal component analysis (PCA) was conducted using the Prcomp function to evaluate batch effects. Batch effect correction was performed using the SVA package. Differential expression analysis was performed using the limma package, applying the screening criteria of *p* < 0.05 and |log₂ fold change (log₂FC)| > 1. The DAVID database (https://david.ncifcrf.gov/) was used to perform Gene Ontology (GO) functional annotation with a significance threshold of *p* < 0.05. The STRING database (https://cn.string-db.org/cgi/input?sessionId=bArbkZvFa3Uw%26input_page_show_search=on) were used to construct a protein-protein interaction (PPI) network for the differentially expressed genes (DEGs). The GEPIA2 database (http://gepia2.cancer-pku.cn) was used to validate the differences in target gene expression between LUAD and normal lung tissue. The KM-plotter database (http://kmplot.com/analysis/index.php?p=service%26cancer=pancancer_rnaseq) and the GEPIA3 (https://gepia3.bioinfoliu.com/) database were used to analyze the association between target genes and overall survival (OS), recurrence-free survival (RFS) and progression-free interval (PFI) in patients with LUAD, thus evaluating their prognostic value. Based on the TCGA-LUAD dataset (https://portal.gdc.cancer.gov/), Gene Set Enrichment Analysis (GSEA) software (version 4.0.3) was used to annotate signaling pathways and identify significantly enriched pathways (false discovery rate [FDR] < 0.25, *p* < 0.05) to explore the potential biological mechanisms of SGPP2 in LUAD. Pan-cancer gene expression matrices were obtained from the UCSC Xena database (https://xenabrowser.net), and correlation analysis between target genes and other genes was performed in R. The upstream transcription factors (TFs) of the *SGPP2* gene were predicted using the PROMO (https://alggen.lsi.upc.es/cgi-bin/promo_v3/promo/promoinit.cgi?dirDB=TF_8.3) and JASPAR (http://jaspar2016.genereg.net/) databases.

### Clinical specimens

Tissue samples were collected from patients diagnosed with LUAD at the First Affiliated Hospital of Nanchang University, including 135 pairs of paraffin-embedded tissues and 6 fresh clinical specimens (tumor and matched adjacent normal tissues). The inclusion criteria were a diagnosis of LUAD confirmed by pathological examination and complete clinical data. Patients who received preoperative chemotherapy, radiotherapy, or chemoradiotherapy were excluded. The study protocol was approved by the Ethics Committee of the First Affiliated Hospital of Nanchang University (Approval No. (2025)CDYFYYLK(09–029)).

### Immunohistochemistry (IHC)

Tissue sections  (4 μm thick) were prepared from paraffin-embedded samples. Immunodetection was performed using the following primary antibodies: SGPP2 (102-23119, Raybiotech, 1:100), β-catenin (T0300R, Immunoway, 1:2000), and Ki67 (A2094, Abclonal, 1:800). Two experienced pathologists independently evaluated the staining results in a double-anonymized manner. The scoring criteria were based on staining area and intensity. Staining area: <25% = 1, 25%–49% = 2, 50%–74% = 3, > 75% = 4; staining intensity: 0 (-), 1 (+), 2 (++), 3 (+++); judgment criteria: The Receiver Operating Characteristic (ROC) curve was plotted for survival outcome and IHC score, and the score corresponding to the maximum Youden index was used as the cut-off value.

### Cell culture and transfection

LUAD cell lines (NCI-H1299, A549, PC9, NCI-H1975), a human bronchial epithelial cell line (BEAS-2B), and a human embryonic kidney cell line (293T) were procured from the Cell Resource Center of the Shanghai Institutes for Biological Sciences, Chinese Academy of Sciences. All cell lines have been identified by cell line identification and tested for mycoplasma. All cell lines were cultured in complete medium supplemented with 10% fetal bovine serum (FBS; 900 − 108, Excell Bio) and maintained in an incubator at 37 °C with 5% CO₂. Two days after transfection with lentiviruses (pLV3-U6-sh1SGPP2(human)-IRES-Puro, pLV3-U6-sh2SGPP2(human)-IRES-Puro, pLV3-CMV-SGPP2(human)-3×flag-Puro, and pLV3-CMV-TFAP2A(human)-HA-Puro), stably transfected cell lines were selected using 2 µg/mL puromycin (P8230, Solarbio). Transient transfection with siRNA and plasmids was performed using the TurboFect Transfection Reagent (R0531, Thermo Fisher Scientific) according to the manufacturer’s instructions. Cells were subjected to subsequent experiments, 48–72 h post-transfection. The sequences of TFAP2A-siRNA and negative control si-NC are listed in Supplementary Table 1.

### Quantitative real-time polymerase chain reaction (qRT-PCR)

Total RNA was extracted from the cells using aTotal RNA Miniprep kit (AP-MN-MS-RNA-250, Axygen). Genomic DNA was removed, and cDNA synthesis was performed using the HiScript II Q RT SuperMix for qPCR (+ gDNA wiper) kit (R223-01, Vazyme). qRT-PCR was performed using ChamQ SYBR qPCR Master Mix (High ROX Premixed) (Q341-02, Vazyme), with *GAPDH* as the internal reference gene. The relative expression levels of target genes were calculated using the 2^-ΔΔCt^ method, and the primer sequences are listed in Supplementary Table 1.

### Western blotting (WB)

Cells or tissues were collected and lysed using RIPA buffer (R0020, Solarbio) supplemented with 1% Protein Phosphatase Inhibitor Cocktail (All-in-one, 100×; P1260, Solarbio) and 1% Phenylmethylsulfonyl Fluoride (PMSF; P0100, Solarbio) for protein extraction. Protein concentration was quantified using a BCA Protein Quantification Kit (E112-02, Vazyme). Subsequently, SDS-PAGE Loading Buffer (P1016, Solarbio) was added, followed by denaturation at 100 °C for 10 min. Equal amounts of protein were separated by SDS-PAGE and transferred onto polyvinylidene fluoride (PVDF) membranes (ISEQ00010, Millipore, Billerica, MA, USA) via a wet transfer. Membranes were blocked with 5% skim milk in TBST buffer at room temperature (RT) for 1 h, then incubated with primary antibodies (GAPDH: 60004-1-Ig, Proteintech, 1:5000; β-actin: 66009-1-Ig, Proteintech, 1:5000; SGPP2: 102-23119, Raybiotech, 1:400; TFAP2A: 13019-3-AP, Proteintech, 1:4000; PCNA: 10205-2-AP, Proteintech, 1:5000; CyclinD1: 26939-1-AP, Proteintech, 1:2000; CyclinB1: 55004-1-AP, Proteintech, 1:1000; MMP9: AF5228, Affinity, 1:1000; E-cadherin: 20874-1-AP, Proteintech, 1:10000; N-cadherin: 22018-1-AP, Proteintech, 1:2000; Vimentin: 10366-1-AP, Proteintech, 1:5000; β-catenin: PT0300R, Immunoway, 1:2000; GSK3β: YM1273, Immunoway, 1:1000; p-GSK3β (Ser9): PT0078R, Immunoway, 1:1000; Histone H3: 17168-1-AP, Proteintech, 1:5000; SREBP1: 14088-1-AP, Proteintech, 1:2000; FASN: PT0554R, Immunoway, 1:4000; SCD1: YN2991, Immunoway, 1:1000; SGPP1: YN4019, Immunoway, 1:1000; SKI1: YT4309, Immunoway, 1:1000) overnight at 4 °C. The next day, the membranes were incubated with goat gnti-rabbit or mouse IgG (H + L) (BS13278/BS12478, Bioworld; 1:20000) at RT for 1 h.

### Cell counting kit-8 (CCK-8) assay

Transfected cells were seeded into 96-well plates at a density of 2.0 × 10³ cells/well. The time at which the cells completely adhered to the plate was defined as day 0 of the experiment. At 0, 1, 2, and 3 days post-adhesion, 90 µL of complete medium supplemented with 10 µL of CCK-8 reagent (BA00208, Bioss) was added to each well via medium replacement. After incubation at 37 °C for 2 h, the optical density (OD) was measured at 450 nm.

### Colony formation assay

Transfected cells were seeded into 6-well plates at a density of 1.5 × 10³ cells/well and cultured statically in a 37 °C, 5% CO₂ incubator for 10–14 days. The culture was terminated when colonies (≥ 50 cells/colony) were formed. The cells were fixed with methanol for 30 min and stained with Crystal Violet Ammonium Oxalate Solution (G1062, Solarbio) for 15 min.

### 5-Ethynyl − 2’- deoxyuridine (EdU) assay

Transfected cells were seeded into 96-well plates at a density of 5.0 × 10³ cells/well. The assay was performed according to the manufacturer’s instructions using the Cell-Light EdU Apollo488 In Vitro Kit (C10310-3, RIBOBIO). The EdU-positive cell rate was calculated using the following formula: (Number of EdU-positive cells / number of DAPI-positive cells) × 100.

### Transwell assay

Transfected cells were resuspended in serum-free medium and adjusted to a concentration of 3.5 × 10⁵ cells/mL. Transwell chambers (353097, BD) were placed in 24-well plates. The upper chambers were either coated or left uncoated with BD Matrigel™ Basement Membrane Matrix (356234, Corning), while the lower chambers were filled with 700 µL of complete medium containing 20% FBS. Subsequently, 200 µL of the cell suspension was added to the upper chamber. After incubation at 37 °C, 5% CO₂ incubator for 36–48 h, the cells were fixed with methanol for 30 min and stained with Crystal Violet Ammonium Oxalate Solution (G1062, Solarbio) for 15 min.

### Wound healing assay

Transfected cells were seeded in 6-well plates and cultured until they reached100% confluency. Three parallel scratches were made perpendicular to the bottom of the well using a 200 µL sterile pipette tip. Images of the same field were captured at 0 and 24 h after scratching. The wound healing rate was calculated using the formula: Healing Rate = (0 h wound area − 24 h wound area) / 0 h wound area × 100.

### Cell cycle assays

Transfected cells were seeded in 6-well plates at a density of 2.0 × 10⁵ cells/well. When the cells reached approximately 70% confluence, they were digested with 0.25% trypsin (T1350, Solarbio), washed twice with cold 1× phosphate buffer saline (PBS), and stained with a working solution (0.5 mL) from the Cell Cycle Assay kit (C543, Dojindo) for flow cytometry analysis.

### Lipid staining

Transfected cells were seeded into 24-well plates containing cell-climbing slices at a density of 5.0 × 10³ cells/well. After cell adhesion and cryosectioning of fresh tissues, lipid droplets were stained according to the protocols of the Oil Red O Staining kit (G1262, Solarbio) and Lipid Fluorescent Staining kit (G1264, Solarbio).

### Detection of intracellular triglyceride (TG) and total cholesterol (TC) levels

Transfected cells were seeded into 6 cm culture dishes at a density of 5.0 × 10⁵ cells/dish and cultured until the logarithmic growth phase. The cells were trypsinized, collected, and washed twice with cold 1× PBS. Subsequently, 150 µL of PBS containing 2% Triton X-100 (9002-93-1, Solarbio) was added to cells on ice for 40 min to lyse the cells. Blank, standard, and sample groups were set up according to the instructions of the Total Cholesterol Assay kit (A111-1-1, Njjcbio) and Triglyceride Assay kit (A110-1-1, Njjcbio). After incubation at 37 °C for 10 min, the OD was measured at 500 nm.

### Enzyme-linked immunosorbent assay (ELISA)

S1P levels in cell lysates were quantified using a commercial S1P ELISA kit (EH2564, Fine Test Biotech).

### Chromatin immunoprecipitation (ChIP) assay

A549 and H1299 cells were seeded in 15 cm culture dishes and cultured until they reached 80% confluence. The ChIP-IT^®^ Express Enzymatic kit (53009, Active Motif) was used for this experiment. Cell fixation, lysis, and enzymatic digestion of the chromatin were performed according to the manufacturer’s instructions. The size of the digested chromatin fragments (200–1000 bp) was verified by agarose gel electrophoresis. Chromatin immunoprecipitation(ChIP) was performed using an antibody specific for TFAP2A (13019-3-AP, Proteintech). Subsequent steps included chromatin elution, reverse cross-linking, and Proteinase K treatment. Using input DNA and IgG control DNA as references, PCR amplification of the *SGPP2* promoter region was performed using the primers listed in Supplementary Table 1. PCR products were separated by 1% agarose gel electrophoresis.

### Dual-luciferase reporter assay

*SGPP2* promoter fragments containing either wild-type (WT) or mutant (MUT) TFAP2A binding sites were amplified and cloned into the pGL3 luciferase reporter vector (Fuzhou Zaiji Biotechnology). Reporter vectors were cotransfected with oe-NC or oe-TFAP2A into 293T cells. After 48 h of transfection, dual-luciferase activity was measured using a Dual Luciferase Reporter Gene Assay kit (11402ES60, Yeasen).

### Co-immunoprecipitation (CoIP)

CoIP was performed in strict accordance with the protocol provided for protein A/G magnetic beads (HY-K0202, MCE). The antibodies utilized in the assay included anti-GFP (50430-2-AP/66002-1-Ig, Proteintech) and anti-HA (51064-2-AP/66006-2-Ig, Proteintech).

### Subcutaneous tumor xenograft model in nude mice

PC9 cells were transfected with either oe-NC or oe-SGPP2 lentiviral vectors. The treated cells were subcutaneously inoculated into BALB/c nude mice (4 weeks old) and designated as the oe-NC and oe-SGPP2 groups, respectively. Tumor volume was measured 3 days post-inoculation and then every 3 days thereafter. When the average tumor diameter approached 15 mm, the mice were euthanized, and the tumor tissue was collected, photographed, and weighed. The animal experiments were approved by the Laboratory Animal Welfare and Ethics Committee of the First Affiliated Hospital of Nanchang University (approval no. CDYFY-IACUC-202505GR094).

### Statistical analysis

All experimental data are presented as mean ± standard deviation (mean ± SD), with error bars in the graphs representing the mean ± standard error of the mean (mean ± SEM). Statistical analyses were performed using SPSS software (version 26.0; IBM, California, USA). The independent samples *t*-test or paired-samples *t*-test was used for comparisons between two groups. One-way analysis of variance (ANOVA) was used for comparisons among three groups, and Bonferroni correction was used for multiple comparisons. The optimal cutoff value for IHC scoring was determined through ROC curve analysis. The association between SGPP2 expression and the clinical characteristics of patients with LUAD was analyzed using the Pearson χ² test. Survival analysis was performed using the Kaplan-Meier method, and survival curves were compared using log-rank tests. Univariate and multivariate Cox regression analyses were performed to identify prognostic factors for patients with LUAD. Statistical graphs were generated using GraphPad Prism software (version 9.0; GraphPad Software, USA). All experiments were independently repeated at least 3 times. Statistical significance was defined as ⁎*p* < 0.05, ⁎⁎*p* < 0.01, ⁎⁎⁎*p* < 0.001.

## Results

### SGPP2 is highly expressed in LUAD and correlates with poor prognosis

**Fig. 1:**
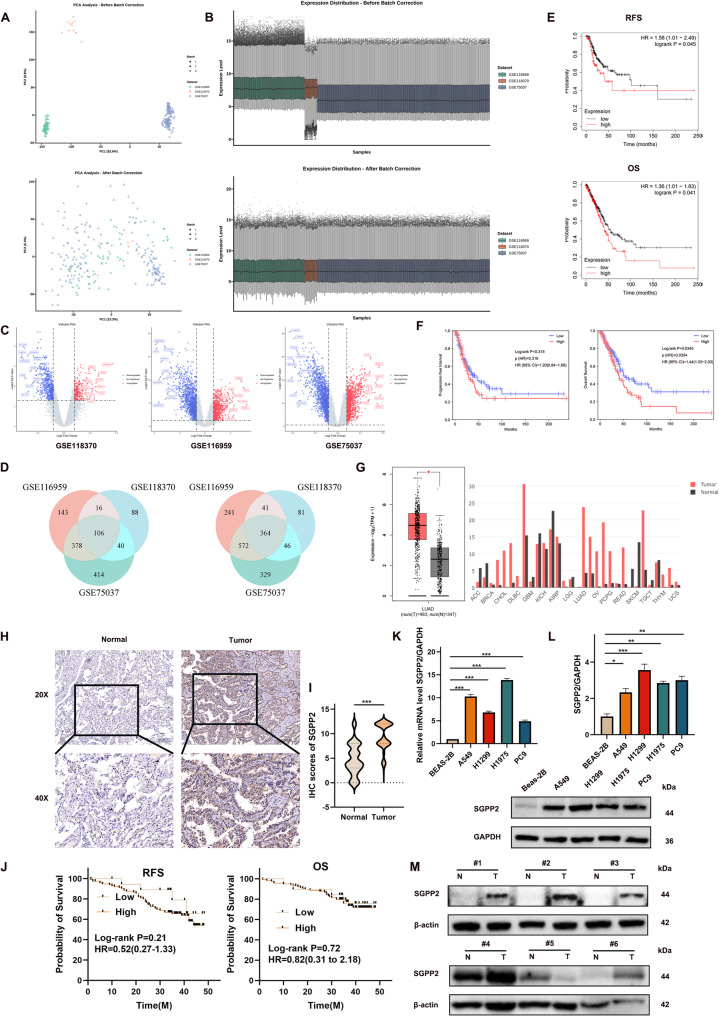
High SGPP2 expression in LUAD patients and its correlation with prognosis. (**A**) PCA plots of three GEO datasets (GSE75037, GSE118370, and GSE116959) before and after batch effect removal. (**B**) Box plots of three GEO datasets before and after batch effect correction. (**C**) Volcano plots of DEGs between LUAD tissues and normal lung tissues from three GEO datasets after batch effect removal (red dots represent upregulated DEGs, blue dots represent downregulated DEGs, and gray dots represent non-DEGs). (**D**) Venn diagram showing the number of common DEGs across the three datasets (intersection indicates 106 commonly upregulated and 364 commonly downregulated DEGs). (**E**) RFS (top) and OS (bottom) curves of LUAD patients stratified by high/low SGPP2 expression in the KM-plotter database. (**F**) PFI (left) and OS (right) curves of LUAD patients stratified according to SGPP2 high/low expression in the GEPIA2 database. (**G**) SGPP2 expression in pan-cancer and LUAD tissues compared to normal tissues (GEPIA2 database). (**H**) IHC staining results of the SGPP2 in lung adenocarcinoma tumor and adjacent normal tissues. (**I**) Quantitative analysis of SGPP2 protein expression in LUAD and adjacent normal tissues. (**J**) Kaplan-Meier survival curves for RFS (left) and OS (right) of LUAD patients with high/low SGPP2 expression (horizontal axis: survival time in months [M]). (**K, L**) Relative SGPP2 expression in BEAS-2B and LUAD cell lines (qRT-PCR and WB). (M) WB analysis of SGPP2 protein expression in six pairs of fresh LUAD tumor tissues (T) and adjacent normal tissues (N). ⁎p < 0.05; ⁎⁎p < 0.01; ⁎⁎⁎p < 0.001.

To identify DEGs between LUAD and normal lung tissues, three independent GEO datasets (GSE75037, GSE118370, and GSE116959) were standardized and preprocessed. The initial validation of the datasets confirmed that all three had undergone prior normalization and log_2_ transformation (Supplementary Fig. 1A–C). Following batch effect correction, sample clustering revealed grouping driven by biological characteristics rather than technical batch effects (Fig. 1A, B). Subsequent differential expression analysis identified 470 common DEGs across all three datasets, including 106 upregulated and 364 downregulated genes (Fig. 1C, D). To investigate potential interactions among these DEGs, a gene list was uploaded into the STRING database to construct a protein–protein interaction (PPI) network. The analysis identified ubiquitin-conjugating enzyme E2 T (UBE2T) and sphingosine-1-phosphate phosphatase (SGPP1/SPP1) as core hub genes in the network (Supplementary Fig. 1D). GO enrichment analysis of differentially expressed genes revealed significant enrichment in the “positive regulation of Wnt signaling pathway” and “positive regulation of PI3K/Akt signal transduction” within the biological process (BP) category, as well as “sphingosine-1-phosphate phosphatase activity” in the molecular function (MF) category (Supplementary Fig. 1E). Validation of *SGPP1* expression in LUAD using the GEPIA2 database showed no statistical significance (Supplementary Fig. 1G). *SGPP2*, a homolog of *SGPP1*, was identified as a key node in the PPI network. Further analysis revealed significantly elevated SGPP2 expression in multiple malignant tumor tissues, including LUAD (Fig. 1G). Moreover, LUAD patients with high *SGPP2* expression had significantly shorter recurrence-free survival (RFS) than those with low expression (hazard ratio [HR] = 1.58, 95% confidence interval [CI]: 1.01–2.49, log-rank *p* = 0.045). Consistently, OS was also significantly shorter in the high SGPP2 expression group than in the low expression group (HR = 1.36, 95% CI: 1.01–1.83, log-rank *p* = 0.041) (Fig. 1E). Subsequently, we performed survival analysis using the GEPIA3 database. The results indicated that the high *SGPP2* expression group exhibited significantly shorter OS compared to the low *SGPP2* expression group (HR = 1.44, 95% CI: 1.03–2.03, Log-rank *p* = 0.0345), suggesting a strong correlation between elevated *SGPP2* expression and poor OS in LUAD patients. However, in the PFI analysis, no statistically significant difference was observed between the survival curves of the high and low *SGPP2* expression groups (HR = 1.20, 95% CI: 0.84–1.83, Log-rank *p* = 0.316) (Fig. 1F). These findings suggest that high *SGPP2* expression may serve as an independent risk factor affecting OS in patients with LUAD, yet its predictive value for tumor recurrence requires further validation.

To validate SGPP2 upregulation and its clinical significance in LUAD, IHC was performed on 135 pairs of paraffin-embedded LUAD tissue samples. These results confirmed that SGPP2 expression was upregulated in LUAD (Fig. 1H, I). SGPP2 levels were significantly higher in LUAD tissues than in adjacent normal tissues (Fig. 1M), consistent with the observations in cell lines (Fig. 1K, L). Among the paraffin-embedded samples, high SGPP2 expression was detected in 116 (85.93%) and low expression in 19 (14.07%) patients. In adjacent normal tissues, low SGPP2 expression was observed in 99 (73.33%) patients, and high expression in 36 (26.67%). The proportion of high SGPP2 expression was significantly higher in tumor tissues than in adjacent normal tissues (*p* = 0.000) (Fig 1I).

Furthermore, SGPP2 expression was significantly correlated with lymph node metastasis (*p* = 0.001) (Table 1). We conducted a systematic analysis of the prognostic factors in patients with LUAD. Univariate Cox regression analysis revealed that M stage (*p* = 0.000, HR = 0.231, 95% CI: 0.112–0.476), clinical stage (*p* = 0.000, HR = 0.197, 95% CI: 0.079–0.489), lymph node metastasis (*p* = 0.041, HR = 0.482, 95% CI: 0.240–0.970), differentiation grade (*p* = 0.012, HR = 4.094, 95% CI: 1.385–12.104), Napsin A + (*p* = 0.008, HR = 0.381, 95% CI: 0.187–0.775), and CK-7+ (*p* = 0.037, HR = 0.470, 95% CI: 0.231–0.955) were all unfavorable prognostic factors. Subsequent multivariate Cox regression analysis confirmed that M stage (*p* = 0.000, HR = 0.245, 95% CI: 0.116–0.520) and Napsin A + (*p* = 0.043, HR = 0.477, 95% CI: 0.233–0.978) were independent risk factors for poor prognosis (Table 2).

However, no statistically significant association was observed between SGPP2 expression and either RFS (log-rank *p* = 0.21; HR = 0.52, 95% CI: 0.27–1.33) or OS (log-rank *p* = 0.72, HR = 0.82, 95% CI: 0.31–2.18) (Fig. 1J). These findings diverge from those of the KM-plotter and GEPIA2 databases analysis, which may be attributable to the relatively small sample size (*n* = 135) and the potential biases inherent in single-center data collection.

**Table 1:** Correlation between SGPP2 expression and clinicopathological characteristics of LUAD patients

Characteristic		SGPP2(*n* = 135)		*p*
		High expression	Low expression	
Sample	Tumor	116	19	0.000
	Normal	36	99	
Gender	Male	64	8	0.290
	Female	63	11	
Age(years)	≥ 56	84	16	0.280
	<56	32	3	
Tumor size(cm)	>3	49	8	0.991
	≤ 3	67	11	
Lymphatic metastasis	Yes	58	3	0.005
	No	58	16	
Vascular tumor thrombus	Yes	11	2	0.890
	No	105	17	
Neural invasion	Yes	5	0	0.356
	No	111	19	
T stage	T1 + T2	102	18	0.382
	T3 + T4	14	1	
N stage	N0 + N1	94	15	0.831
	N2 + N3	22	4	
M stage	M0	74	16	0.080
	M1	42	3	

### SGPP2 promotes LUAD progression

**Fig. 2:**
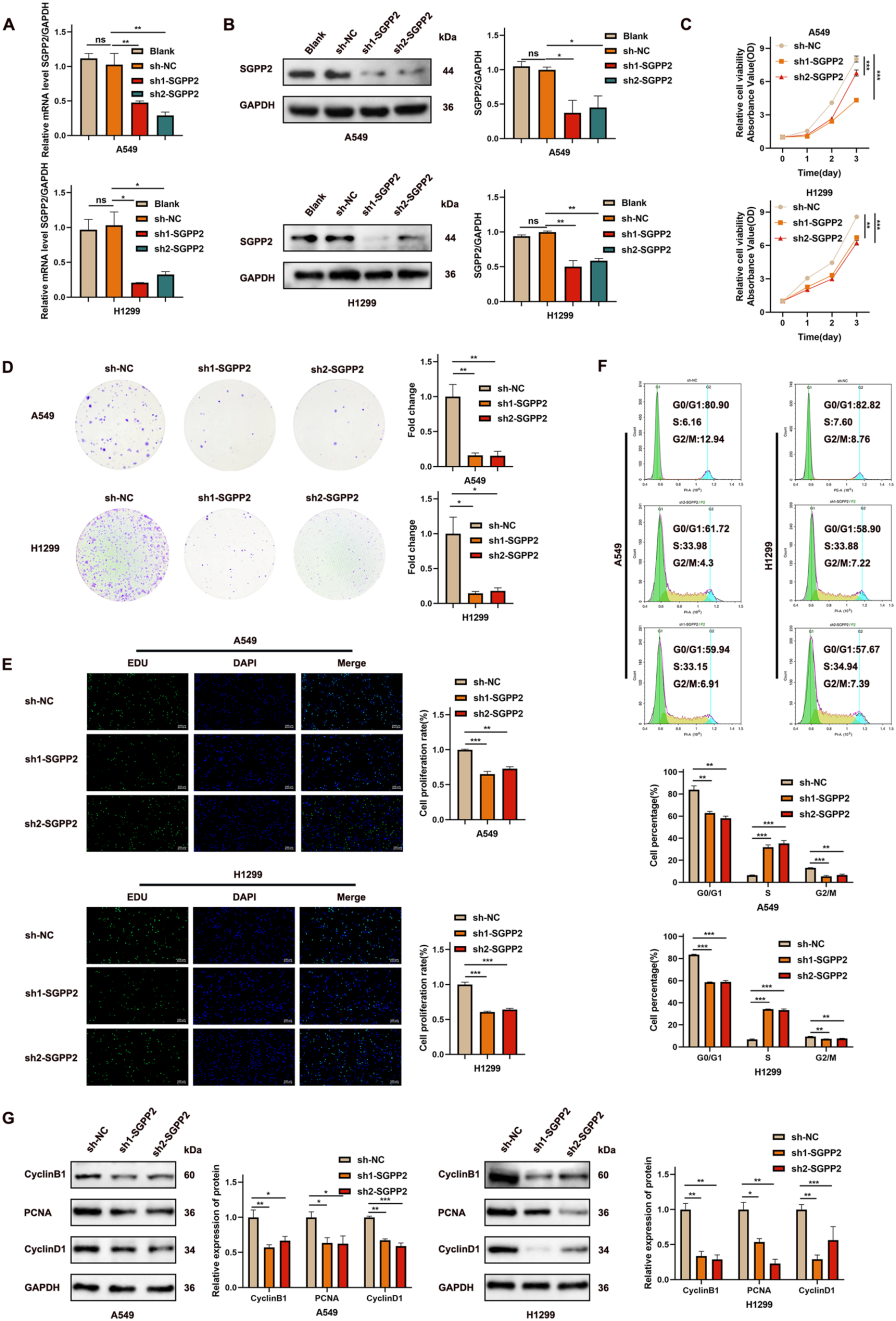
SGPP2 knockdown suppresses LUAD cell proliferation. (**A**, **B**) Validation of SGPP2 knockdown efficiency in A549 and H1299 cells (qRT-PCR and WB). (**C**–**E**) Effect of SGPP2 knockdown on cell proliferation (CCK-8, colony formation, and EdU assays). (**F**) Effect of SGPP2 knockdown on cell cycle distribution (flow cytometry analysis). (**G**) WB analysis of cell cycle-related proteins after SGPP2 knockdown. ⁎*p* < 0.05; ⁎⁎*p* < 0.01; ⁎⁎⁎*p* < 0.001

Stable SGPP2 knockdown cell lines were established using A549 and H1299 cells via lentiviral transfection, and an SGPP2-overexpressing cell line was constructed using the PC9 cells line. Transfectionefficiency was validated by qRT-PCR and WB (Fig. 2A, B; Supplementary Fig. 2A, B).

**Table 2:** Univariate and multivariate analysis were used to analyze the influencing factors of LUAD

Variable		*n* = 135	Univariable Analysis			Multivariable Analysis		
			*p*-Value	HR	95% CI	*p*-Value	HR	95% CI
Gender	Male/Female	72/63	0.877	0.877	0.443–1.737			
Age(years) ≥ 56	Yes/No	100/35	0.770	0.888	0.400–1.969			
Family history	Yes/No	3/132	0.736	0.696	0.095–5.099			
Smoking history	Yes/No	65/70	0.974	1.012	0.510–2.008			
T stage	T1/T2/T3/T4	79/41/9/6	0.134	0.483	0.186–1.252			
N stage	N0/N1/N2/N3	77/32/24/2	0.050	0.501	0.251–1.000			
M stage	M0/M1	90/45	0.000	0.231	0.112–0.476	0.000	0.245	0.116–0.520
Clinical stages	I/II/III/IV	49/18/23/45	0.000	0.197	0.079–0.489			
Tumor size≥3 cm	Yes/No	57/78	0.775	1.107	0.511–2.226			
Lymphatic metastasis	Yes/No	61/74	0.041	0.482	0.240–0.970			
Vascular tumor thrombus	Yes/No	13/122	0.489	0.691	0.243–1.968			
Neural invasion	Yes/No	5/130	0.497	0.609	0.146–2.547			
Grade of differentiation	Low/Medium/High	47/54/24	0.012	4.094	1.385–12.104			
TTF-1+	Yes/No	33/102	0.054	0.498	0.245–1.012			
NaspinA+	Yes/No	28/107	0.008	0.381	0.187–0.775	0.043	0.477	0.233–0.978
CK-7+	Yes/No	32/102	0.037	0.470	0.231–0.955	0.181	0.392	0.099–1.545
VEGF+	Yes/No	77/58	0.362	1.374	0.694–2.724			
IHC score of SGPP2 ≥ 8	Yes/No	116/19	0.718	0.821	0.290–2.346	0.730	1.254	0.316–4.983

Note: TFF-1: Thyroid Transcription Factor-1; CK-7: Cytokeratin 7; VEGF: Vascular endothelial growth factor

To evaluate the effect of SGPP2 on LUAD cell proliferation, we performed a CCK-8 assay. The results showed that SGPP2 knockdown significantly reduced the OD, indicating the inhibition of cell proliferation (Fig. 2C). Colony formation and EdU assays further confirmed that SGPP2 growth factor.knockdown impaired clonogenic capacity and DNA replication activity (Fig. 2D, E). In contrast, SGPP2 overexpression enhanced the proliferative capacity (Supplementary Fig. 2C–E). These results suggested a positive correlation between SGPP2 expression and LUAD cell proliferation.

Cell cycle analysis using flow cytometry revealed that SGPP2 knockdown induced S-phase arrest (Fig. 2F), whereas SGPP2 overexpression increased the proportion of cells in the G0/G1 phase and decreased the S-phase population (Supplementary Fig. 2F). WB analysis of cell cycle-related proteins showed that SGPP2 knockdown downregulated CyclinD1, PCNA, and CyclinB1 expression (Fig. 2G), whereas SGPP2 overexpression upregulated their expression (Supplementary Fig. 2G). These results indicate that SGPP2 knockdown suppresses LUAD cell proliferation by inducing S-phase arrest.

**Fig. 3:**
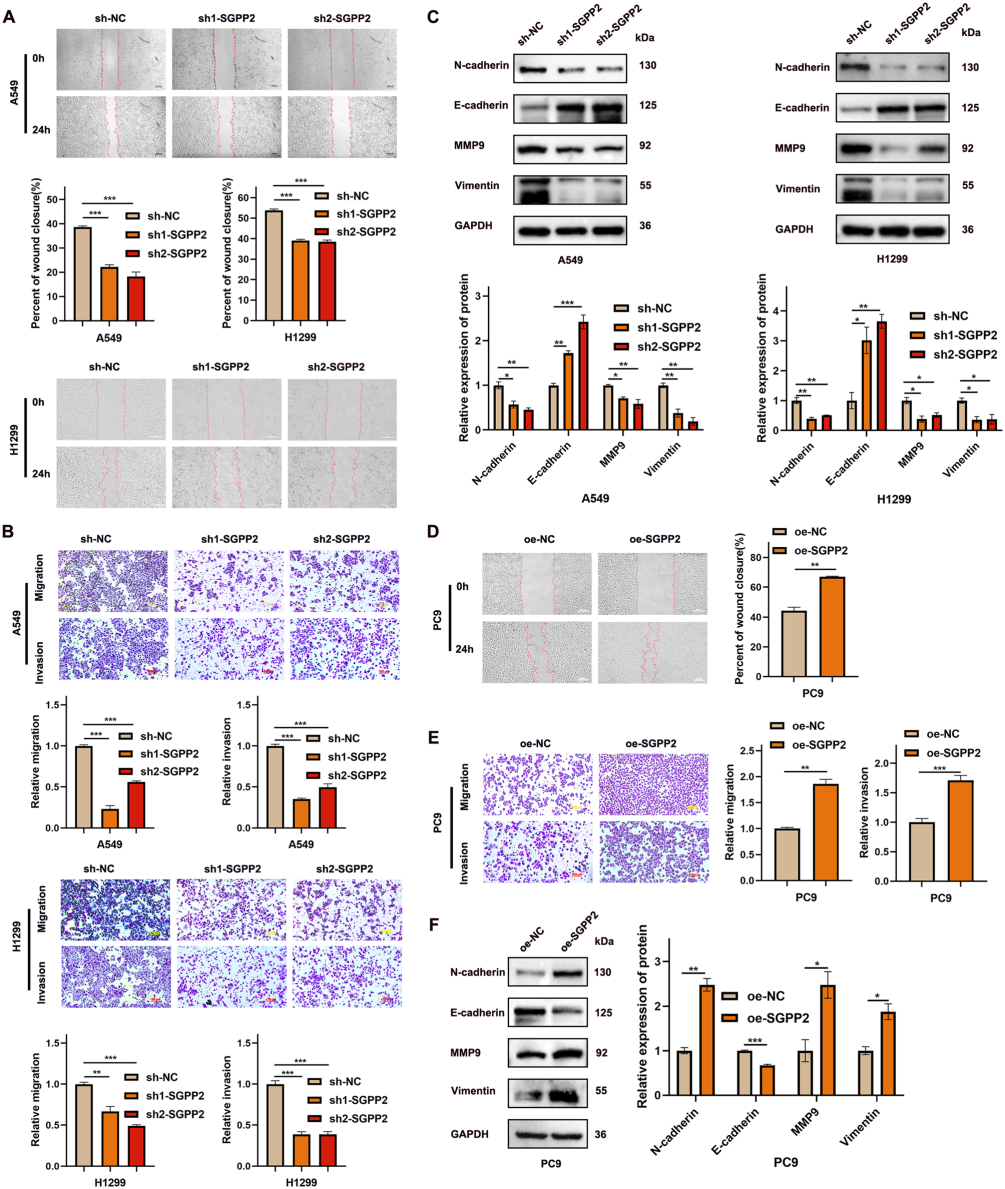
SGPP2 expression modulates LUAD cell migration. (**A**, **B**, **D**, **E**) Effect of SGPP2 knockdown or overexpression on cell migration (wound healing and transwell migration and invasion assays). (**C**, **F**) WB analysis of EMT-related proteins (N-cadherin, MMP9, vimentin, and E-cadherin) after SGPP2 knockdown or overexpression. ⁎*p* < 0.05; ⁎⁎*p* < 0.01; ⁎⁎⁎*p* < 0.001

SGPP2 expression levels were significantly correlated with lymph node metastasis. We further explored the relationship between SGPP2 and the expression of proteins involved in epithelial-mesenchymal transition (EMT). The results showed that SGPP2 knockdown decreased the expression of N-cadherin, MMP9, and vimentin and increased E-cadherin expression, whereas SGPP2 overexpression had the opposite effect (Fig. 3C, F), indicating that SGPP2 promotes EMT in LUAD. Furthermore, wound healing and transwell assays demonstrated that SGPP2 knockdown reduced cell migration and invasion, whereas SGPP2 overexpression enhanced these processes (Fig. 3A, B, D, E).

### High SGPP2 expression activates the Wnt/β-catenin signaling pathway

**Fig. 4:**
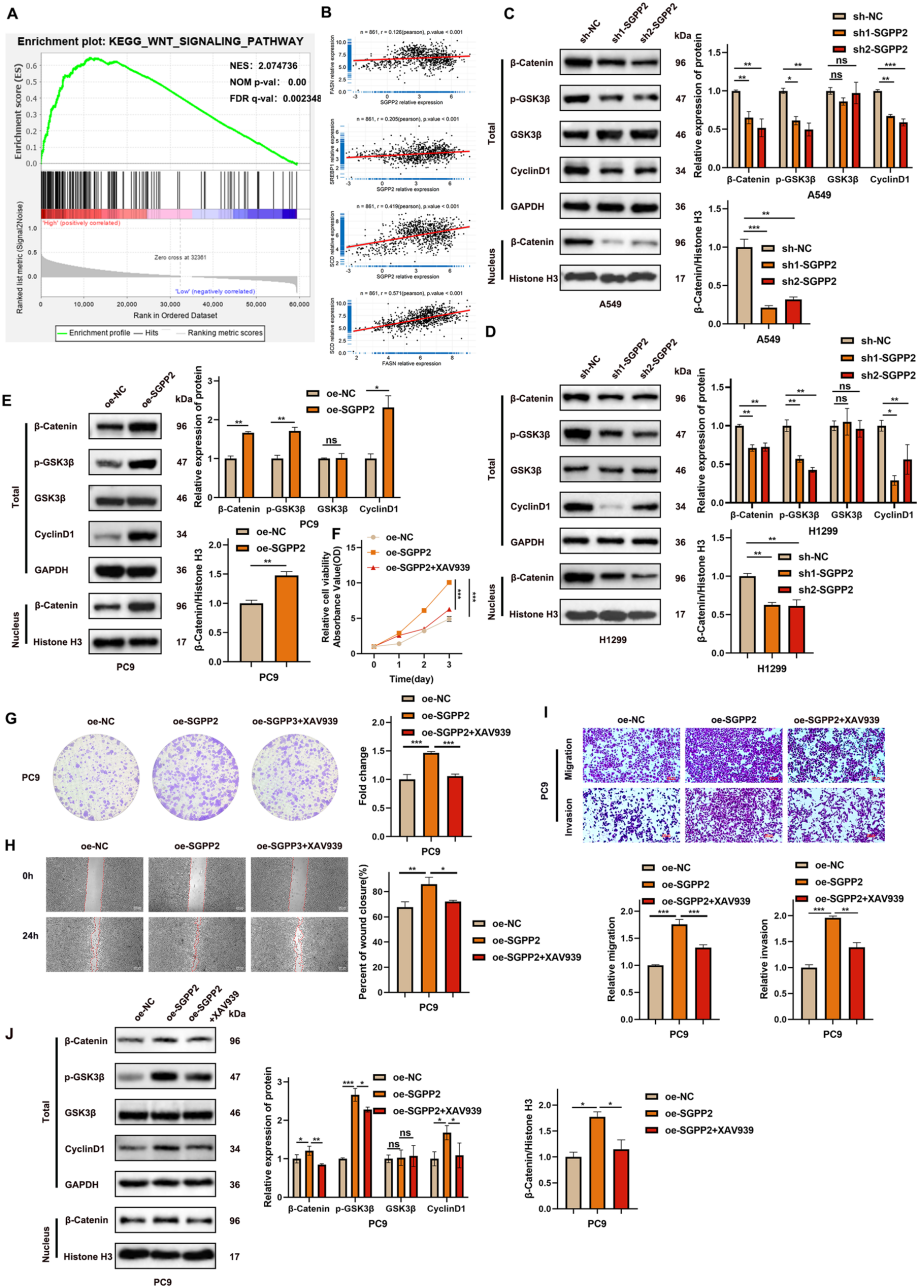
SGPP2 promotes LUAD progression via the Wnt/β-catenin signaling pathway. (**A**) GSEA enrichment analysis showing SGPP2 enrichment in the Wnt signaling pathway. (**B**) Scatter plots showing the correlation between SGPP2 expression and SREBP1, FASN, or SCD1 expression, and between SCD1 and FASN expression. (**C**–**E**) WB analysis of key molecules in the Wnt/β-catenin signaling pathway after SGPP2 knockdown and overexpression. (**F**–**G**) Effect of XAV939 on the proliferation of SGPP2-overexpressing cells (CCK-8 and colony formation assays). (**H**–**I**) Effect of XAV939 on the migration and invasion of SGPP2-overexpressing cells (wound healing assay, transwell migration and invasion assay). (**J**) WB validation demonstrated that XAV939 partially reversed Wnt/β-catenin signaling pathway activation induced by SGPP2 overexpression. ⁎*p* < 0.05; ⁎⁎*p* < 0.01; ⁎⁎⁎*p* < 0.001

GSEA revealed that SGPP2 was significantly enriched in the Wnt signaling pathway (Fig. 4A). GO enrichment analysis revealed that the DEGs were significantly enriched in the Wnt signaling pathway (Supplementary Fig. 1D). Further to explore the role of SGPP2 in this pathway, WB was used to detect the expression of β-catenin, GSK3β, phosphorylated GSK3β (Ser9) (p-GSK3β), and the downstream effector CyclinD1. In the SGPP2 knockdown group, compared with the control group, p-GSK3β levels decreased, while total GSK3β protein levels remained unchanged. Both total β-catenin and nuclear β-catenin levels were significantly reduced, accompanied by a decrease in CyclinD1 expression (Fig. 4C, D). In contrast, in the SGPP2 overexpression group, p-GSK3β levels increased, total GSK3β remained stable, and both total and nuclear β-catenin levels were elevated, along with increased CyclinD1 expression (Fig. 4E). These results indicate that SGPP2 regulates the Wnt/β-catenin signaling pathway, thereby modulating cell proliferation.

To further validate whether SGPP2 regulates the malignant phenotype of LUAD via this pathway, the Wnt/β-catenin signaling pathway inhibitor XAV939 was applied to the SGPP2-overexpressing cell lines. CCK-8 and colony formation assays showed that XAV939 treatment reversed the stimulatory effects of SGPP2 overexpression on cell proliferation. Similarly, wound healing, transwell migration and invasion assays indicated that XAV939 significantly attenuated the enhanced migration induced by SGPP2 overexpression (Fig. 4F–I). Western blot analysis demonstrated that XAV939 reversed SGPP2 overexpression-induced phosphorylation of GSK3β and attenuated nuclear translocation of β-catenin (Fig. 4J).

### SGPP2 promotes lipid biosynthesis in LUAD cells

GSEA revealed that SGPP2 was significantly involved in lipid metabolism in patients with LUAD (Supplementary Table 2). SKI1 activates SREBP1 in the Golgi apparatus via proteolysis, and SREBP1 regulates the expression of FASN and SCD, which is key transcription factions involved in lipid synthesis. FASN and SCD contribute to lipid substrate supply to tumor cells by catalyzing fatty acid synthesis and the production of unsaturated fatty acid, respectively [23, 24].

**Fig. 5:**
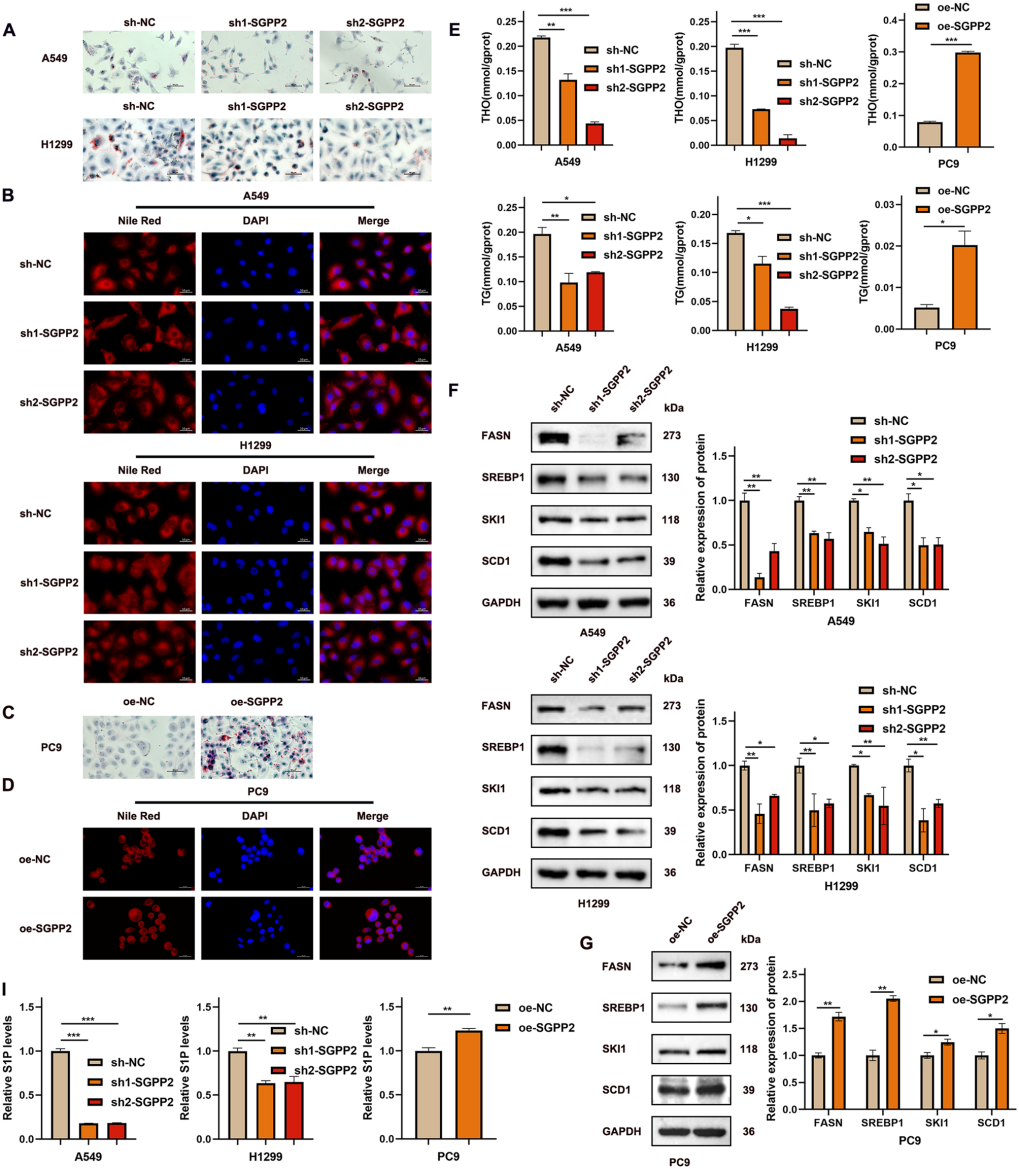
SGPP2 modulates intracellular lipid accumulation and distribution in LUAD cells. (**A**, **C**) Oil Red O staining showing the effect of SGPP2 knockdown or overexpression on intracellular lipid droplet accumulation. (**B**, **D**) Nile Red fluorescence staining showing the effect of SGPP2 knockdown or overexpression on intracellular lipid droplet distribution. (**E**) Quantitative analysis of intracellular TC and TG levels after SGPP2 knockdown and overexpression. (**F**, **G**) WB analysis was performed to examine the impact of SGPP2 expression on lipid metabolism-related proteins; (**I**) Effect of SGPP2 on S1P levels in cell lysates. ⁎*p* < 0.05; ⁎⁎*p* < 0.01; ⁎⁎⁎*p* < 0.001

Gene correlation analysis further showed that SGPP2 expression was significantly and positively correlated with SREBP1, FASN, and SCD in LUAD. A positive correlation between SCD and FASN was observed (Fig. 4B). To explore the functional role of SGPP2 in lipid metabolism, intracellular TC and TG levels were quantified. Oil Red O and Nile Red fluorescent staining were used to visualize lipid droplet accumulation and distribution. The results showed that changes in lipid staining intensity and TC and TG levels were consistent with the alterations in lipid metabolism-related proteins. Compared to the control group, SGPP2 knockdown significantly reduced intracellular TC and TG levels (Fig. 5E), increased lipid droplet aggregation volume, and enhanced lipid droplet accumulation around the cell membrane (Fig. 5A–D). Concurrently, S1P levels in cell lysates were decreased (Fig. 5I), and lipid metabolism-related proteins (SKI1, SREBP1, FASN, SCD1) were downregulated (Fig. 5F). In contrast, the SGPP2 overexpression group showed the opposite trend (Fig. 5G, I). These findings suggest that SGPP2 promotes lipid synthesis in LUAD cells.

### Exogenous S1P can promote cell proliferation, migration and invasion, and activate the Wnt/β-catenin signaling pathway

**Fig. 6:**
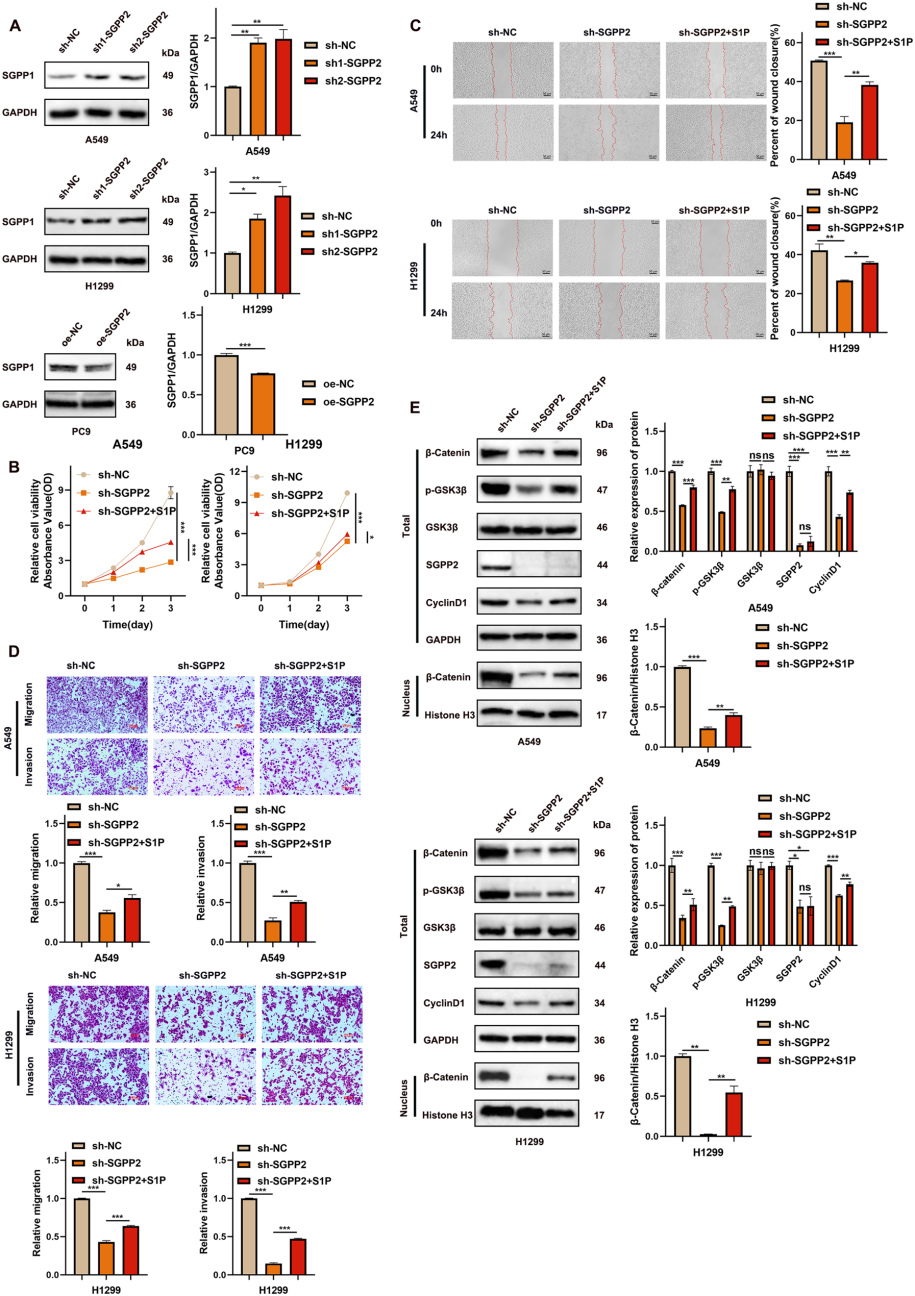
S1P promotes LUAD cell proliferation, migration, and invasion via the Wnt/β-catenin signaling pathway. (**A**) Regulation of SGPP2 expression affects the expression level of its homologous protein SGPP1; (**B**) CCK-8 assay demonstrates that exogenous S1P reverses the suppressive effect of SGPP2 knockdown on LUAD cell proliferation; (**C**, **D**) Wound healing and transwell assays confirm that exogenous S1P reverses the inhibitory effects of SGPP2 knockdown on LUAD cell migration and invasion capabilities; (**E**) Exogenous S1P counteracts the suppressive impact of SGPP2 knockdown on the Wnt/β-catenin signaling pathway

As a specific hydrolytic phosphatase of S1P, SGPP2 regulates S1P production through changes in its expression level, which contradicts its molecular function. To elucidate the mechanisms underlying this phenomenon, we examined the SGPP1 protein expression, a homolog of SGPP2 that also functions as a phosphate hydrolase for S1P. These findings indicate that SGPP2 knockdown led to a significant upregulation of SGPP1 expression compared to the control group, whereas SGPP2 overexpression resulted in a marked downregulation of SGPP1 expression relative to the controls (Fig. 6A).

Further addition of exogenous S1P partially reversed the inhibitory effects of SGPP2 knockdown on cell proliferation, migration, and invasion (Fig. 6B–D). SGPP2 promotes malignant phenotype by modulating S1P levels. Based on this finding, we hypothesized that SGPP2 regulates the Wnt/β-catenin signaling pathway through the S1P signaling axis, thereby promoting LUAD progression. To validate this hypothesis, exogenous S1P was introduced into the SGPP2-knockdown cell models. These results indicate that exogenous S1P partially reversed the inhibitory effect of SGPP2 knockdown on the Wnt/β-catenin signaling pathway (Fig. 6E).

### TFAP2A transcriptionally activates SGPP2

**Fig. 7:**
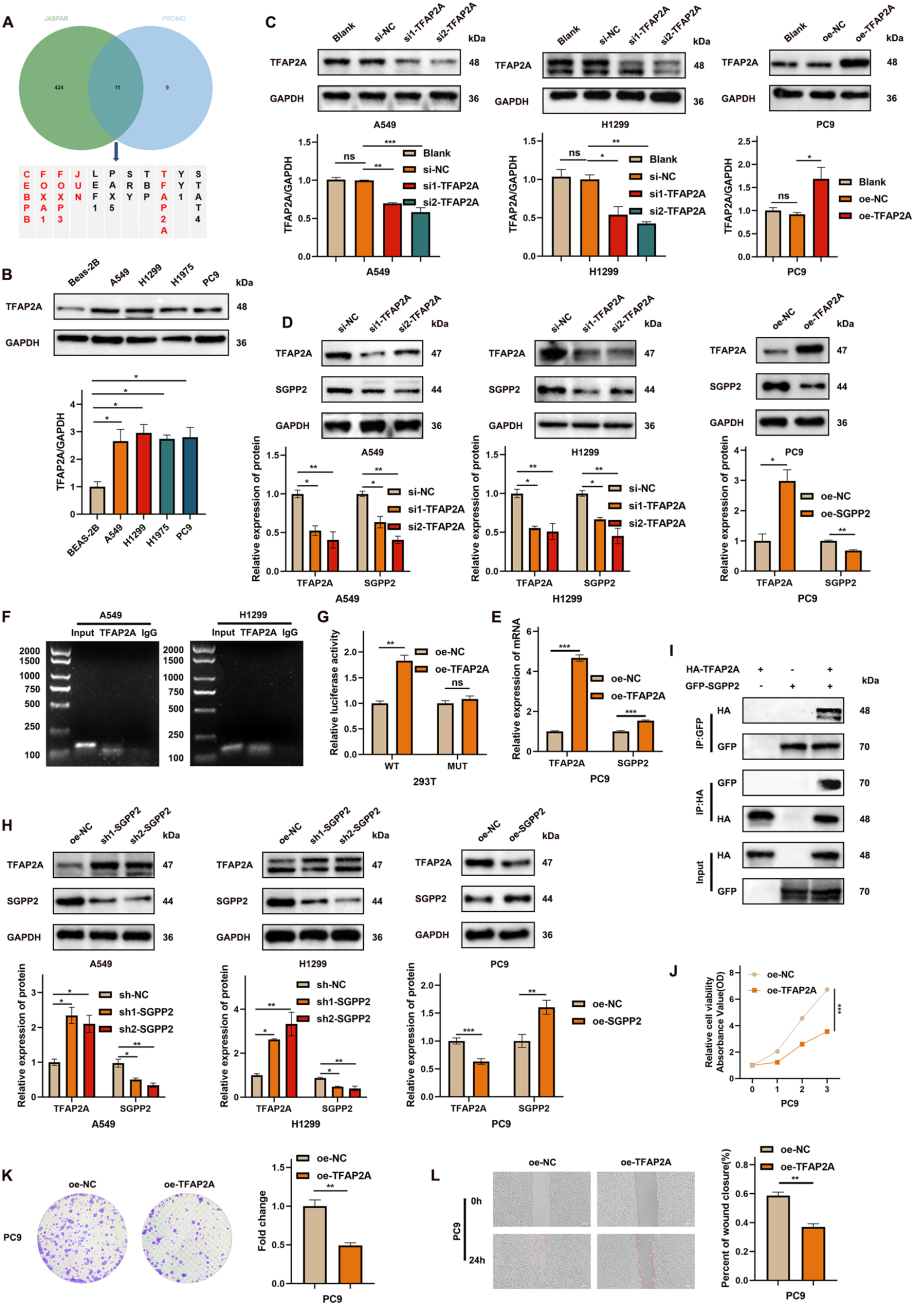
TFAP2A binds to the *SGPP2* promoter and transcriptionally regulates SGPP2 expression in LUAD cells. (**A**) Prediction of upstream TFs of SGPP2 using the JASPAR and PROMO databases. (**B**) WB validation of TFAP2A overexpression in the LUAD cell line. (**C**) Validation of TFAP2A knockdown and overexpression efficiencies. (**D**) WB analysis of SGPP2 expression after TFAP2A knockdown and overexpression. (**E**) qRT-PCR analysis of SGPP2 mRNA expression after TFAP2A overexpression. (**F**) ChIP assay showing the binding of TFAP2A to the *SGPP2* promoter (agarose gel electrophoresis; TFAP2A: TFAP2A antibody group, IgG: negative control group). (**G**) Dual-luciferase reporter assay showing the binding of TFAP2A to the *SGPP2* promoter. (**H**) WB analysis of TFAP2A expression following the knockdown and overexpression of SGPP2. (**I**) Exogenous CoIP assay confirmed the PPI between SGPP2 and TFAP2A. (**J**, **K**) Effect of TFAP2A overexpression on LUAD cell proliferation (CCK-8, and colony formation assays). (**N**, **O**) Effect of TFAP2A overexpression on LUAD cell migration and invasion (wound healing, and transwell assay). ⁎*p* < 0.05; ⁎⁎*p* < 0.01; ⁎⁎⁎*p* < 0.001

The upstream TFs of SGPP2 were predicted using the PROMO and JASPAR databases, and 11 common candidates were identified via intersection analysis (Fig. 7A). Expression analysis using the GEPIA2 database showed that five of these TFs (CEBPB, FOXA1, FOXP3, JUN, and TFAP2A) were differentially expressed between LUAD tumor tissues and adjacent normal tissues (Supplementary Fig. 1H). Subsequent correlation analyses demonstrated an association between all five TFs and SGPP2 expression (Supplementary Fig. 1I). Survival analysis indicated that only CEBPB and TFAP2A significantly affected the LUAD prognosis (Supplementary Fig. 1J). A review of relevant studies revealed that CEBPB does not affect LUAD cell proliferation in vitro or in vivo [25]; therefore, TFAP2A was selected for further research.

First, WB analysis verified that TFAP2A was highly expressed in LUAD cell lines (Fig. 7B). TFAP2A was knocked down using siRNA and overexpressed using a lentivirus, and the effects were validated by WB (Fig. 7C). WB analysis showed that TFAP2A knockdown suppressed the expression of TFAP2A and SGPP2. In contrast, TFAP2A overexpression inhibited SGPP2 expression (Fig. 7D). Subsequent qRT-PCR experiments confirmed that TFAP2A promotes SGPP2 transcription at the mRNA level (Fig. 7E).

To validate the interaction between TFAP2A and SGPP2, ChIP and dual-luciferase reporter assays were performed. The results showed that SGPP2 was significantly enriched in the TFAP2A group compared to the IgG control group (Fig. 7F). 293T cells co-transfected with oe-TFAP2A and SGPP2-WT showed significantly higher luciferase activity than that in the control group. In contrast, no significant change in luciferase activity was observed when 293T cells were co-transfected with oe-TFAP2A and SGPP2-MUT (Fig. 7G).

Subsequently, we performed a gradient transfection experiment using the TFAP2A overexpression plasmid. The results indicated that cell viability was maintained when the plasmid transfection amount ranged from 0.5 to 4 µg, whereas cell death began to occur at or above 6 µg. Within the concentration range tolerated by the cells, TFAP2A significantly promoted the expression of SGPP2 (Supplementary Fig. 3A). To investigate the mechanism underlying the bidirectional regulatory relationship between SGPP2 and TFAP2A, we examined the expression of TFAP2A following alterations in SGPP2 expression levels. The results demonstrated that SGPP2 knockdown led to upregulation of TFAP2A expression, whereas SGPP2 overexpression led to downregulation of TFAP2A expression (Fig. 7H). Further validation via an exogenous co-immunoprecipitation (CoIP) assay confirmed a direct PPI between TFAP2A and SGPP2 (Fig. 7I).

### TFAP2A promotes LUAD progression by regulating SGPP2 expression

**Fig. 8:**
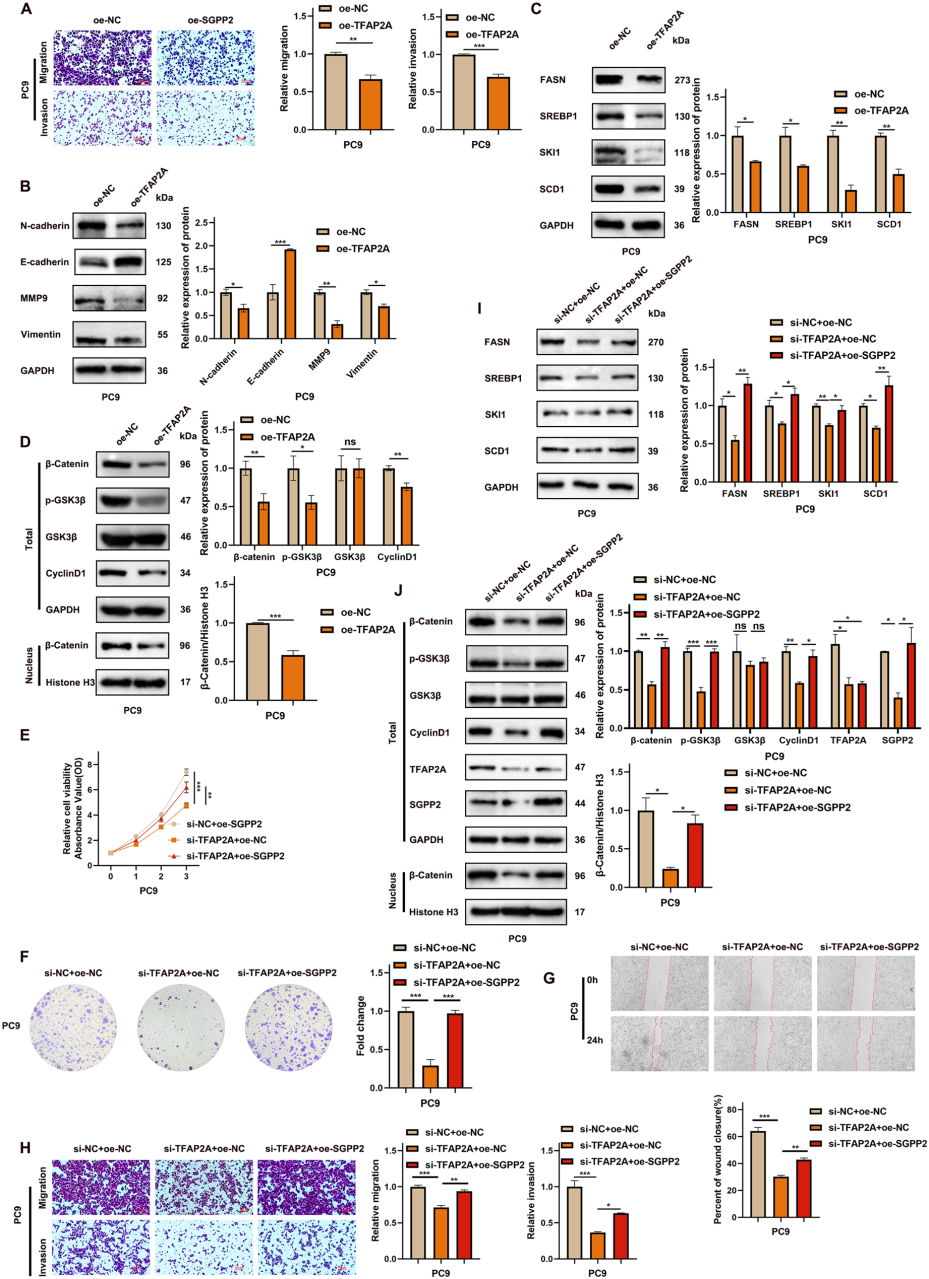
TFAP2A activates the Wnt/β-catenin signaling pathway via SGPP2 and promotes the malignant phenotype of LUAD cells. (**A**) The transwell assay was used to assess alterations in the migratory capacity of LUAD cells following TFAP2A overexpression. (**B**–**D**) WB analysis of EMT-related proteins, key molecules in the Wnt/β-catenin signaling pathway, and lipid metabolism-related proteins after TFAP2A overexpression. (**E**, **F**) Effects of TFAP2A and SGPP2 on LUAD cell proliferation (CCK-8, and colony formation assays). (**G**, **H**) Effects of TFAP2A and SGPP2 on LUAD cell migration and invasion (wound healing, and transwell assays). (**I**) WB analysis of lipid metabolism-related proteins after TFAP2A knockdown and SGPP2 overexpression. (**J**) WB analysis of Wnt/β-catenin signaling pathway-related proteins following TFAP2A knockdown and SGPP2 overexpression. ⁎*p* < 0.05; ⁎⁎*p* < 0.01; ⁎⁎⁎*p* < 0.001

Experimental results showed that SGPP2 promotes the malignant phenotype of LUAD, and TFAP2A can transcriptionally upregulate SGPP2, which, in turn, feeds back to the expression of TFAP2A. However, our results indicated that overexpression of TFAP2A in PC9 cells via lentiviral transfection reduced the suppression of SGPP2 expression. Proliferation and migration assays showed that TFAP2A overexpression suppressed LUAD cell proliferation, migration, and invasion (Figs. 7J–L and 8A). WB analysis further indicated that TFAP2A overexpression inhibited EMT, the Wnt/β-catenin signaling pathway, and lipid accumulation in LUAD cells (Fig. 8B–D). These results suggest that TFAP2A promotes LUAD progression within a specific expression range, whereas excessive overexpression inhibits LUAD progression.

In summary, TFAP2A promotes *SGPP2* transcription in LUAD. SGPP2 overexpression counteracted the suppressive effects of siTFAP2A on SGPP2 expression (Fig. 8J). Compared to the control group, TFAP2A knockdown inhibited LUAD cell proliferation, migration, and invasion, whereas SGPP2 overexpression partially reversed these effects (Fig. 8E–H). Additionally, TFAP2A downregulation reduced intracellular lipid accumulation compared to that in the control group, and this effect was partially rescued by SGPP2 overexpression (Fig. 8I). TFAP2A knockdown inactivated the Wnt/β-catenin signaling pathway, and the restoration of SGPP2 expression significantly attenuated this suppression (Fig. 8J), indicating that the TFAP2A/SGPP2 axis is involved in the regulation of this pathway. In conclusion, silencing TFAP2A suppressed the malignant phenotype of LUAD by modulating SGPP2 expression.

### Overexpression of SGPP2 promotes the growth of xenograft tumors in vivo

**Fig. 9:**
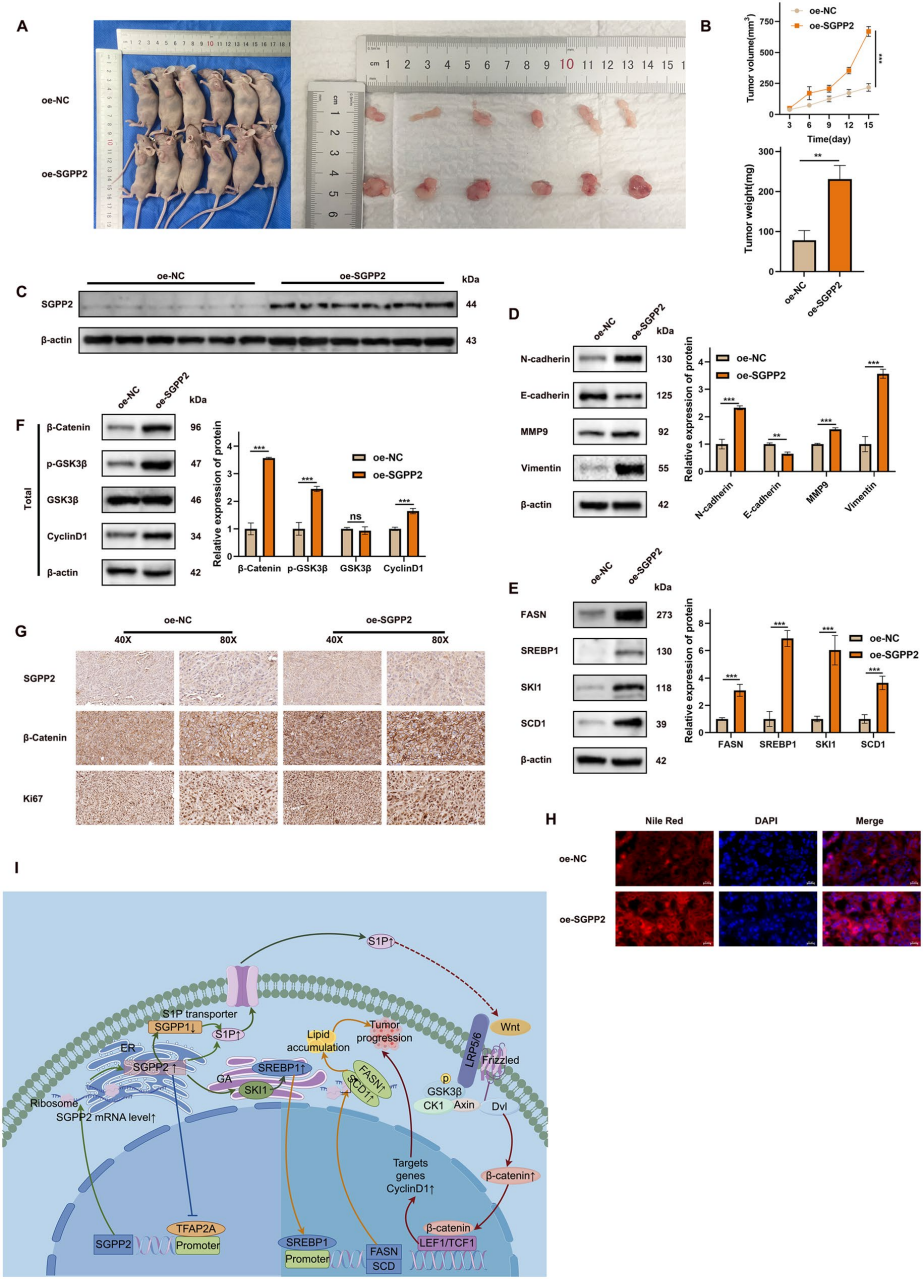
Overexpression of SGPP2 promotes xenograft growth in vivo. (**A**) Subcutaneous xenograft model in nude mice; (**B**) Quantitative analysis of transplanted tumor weight and tumor volume-growth dynamic curve in control group and SGPP2 overexpression group; (**C**) WB was used to detect the expression of SGPP2 in the transplanted tumor tissues of nude mice. (**D**) Western blotting was used to detect E-cadherin, N-cadherin, MMP9, and vimentin in transplanted tumor tissues. (**E**) Western blot was used to detect lipid metabolism-related proteins in transplanted tumor tissues. (**F**) WB was used to detect Wnt/β-catenin signaling pathway-related proteins in xenograft tumor tissues; (**G**) IHC was used to detect Ki-67 and β-catenin expression levels in transplanted tumor tissues. (**H**) Nile red fluorescence staining was used to observe the effect of SGPP2 overexpression on lipid accumulation in transplanted tumors. (**I**) Schematic representation of the molecular mechanism by which SGPP2 regulates LUAD development and progression. ⁎*p* < 0.05; ⁎⁎*p* < 0.01; ⁎⁎⁎*p* < 0.001

We used a subcutaneous xenograft tumor model in nude mice to investigate the biological function of SGPP2 in tumor growth in vivo. These results demonstrated that SGPP2 overexpression significantly enhanced tumor growth after transplantation. The tumor-promoting effect of SGPP2 overexpression was time-dependent, and tumor weight in the oe-SGPP2 group was markedly increased compared to that in the control group (Fig. 9A, B). WB analysis demonstrated that SGPP2 promotes EMT, lipid accumulation, and activation of the Wnt/β-catenin signaling pathway in vivo (Fig. 9D–F). IHC analysis revealed that, relative to the control group, the expression levels of SGPP2, Ki-67, and β-catenin were significantly upregulated in the oe-SGPP2 group (Fig. 9G). Additionally, lipid accumulation in the tumor tissues of the oe-SGPP2 group was significantly higher than that in the control group (Fig. 9H).

## Discussion

Lung adenocarcinoma (LUAD), a malignant tumor subtype with the highest global incidence and mortality, is characterized by insidious onset, rapid progression, and strong therapeutic resistance. Therefore investigating the underlying molecular mechanisms is a critical focus of cancer research [2, 3, 26–28]. Omics analysis provides important technical support for the analysis of the mechanism of disease occurrence and development [29–32]. The interactive regulation of tumor metabolic reprogramming and aberrant signaling pathway activation are core driver of malignant tumor progression [33]. Tumor metabolic reprogramming provides energy and membrane structural precursors to cancer cells, whereas signaling pathways regulate cell proliferation and stemness maintenance. The synergistic interaction between these processes is closely associated with tumor resistance to therapy, recurrence, and metastasis [34].

Sphingolipid metabolism, a key branch of lipid metabolism, contributes to tumor progression via the critical intermediate metabolite S1P, which regulates cell survival, migration, and signaling pathway activation [35, 36]. Previous studies have shown that low SGPP2 expression in colorectal cancer tissues leads to S1P accumulation and is correlated with poor prognosis [37]. In gastric cancer, NUDT21 promotes tumor proliferation and metastasis by positively regulating SGPP2 [10]. SGPP2 exhibits divergent biological functions across breast cancer subtypes [38–40], and one study reported its high expression in LUAD [41]. However, its precise mechanistic role remains unclear.

In the present study, multidimensional validation showed that SGPP2 expression was significantly upregulated in patients with LUAD. More importantly, high SGPP2 expression was closely associated with malignant clinicopathological features and poor prognosis in patients with LUAD. Although no significant difference in OS was observed, potentially due to the limited sample size from a single center and potential biases, consistent findings across multiple databases confirmed SGPP2 as a potential prognostic biomarker for LUAD. This result contrasts sharply with the tumor-suppressive role of SGPP2 in colorectal cancer, suggesting that its function is cancer type-specific. The divergent roles of SGPP2 across malignancies may be attributed to context-specific factors, including the TME, metabolic networks, and signaling pathways.

Functional cell assays confirmed that SGPP2 promotes LUAD cell proliferation, migration, invasion and EMT. SGPP2 enhances LUAD cell migration by regulating the EMT process, which is consistent with the clinical finding that high SGPP2 expression correlates with lymph node metastasis in LUAD patients. These results further confirm the critical role of SGPP2 in LUAD invasion and metastasis.

This study revealed a paradoxical cell cycle phenotype: despite SGPP2 being an oncogene in LUAD, SGPP2 knockdown decreased CyclinD1 and CyclinB1 levels, with no expected accumulation in the G0 + G1 phase. Instead, a reduction in the G0 + G1 population and an increase in the S-phase fraction were observed. In contrast, SGPP2 overexpression produced the opposite phenotype. This observation does not refute the pro-oncogenic role of SGPP2 but rather reflects the cross-regulatory interplay between the S1P metabolic network and cell cycle signaling pathways. SGPP2 knockdown reduces S1P levels, subsequently inhibiting the Wnt/β-catenin pathway. Since β-catenin directly regulates CyclinB1 transcription, pathway suppression results in diminished CyclinB1 expression. This impairment prevents cells that have entered S-phase from completing the G2→M transition, leading to their accumulation in S-phase [42]. SGPP2 knockdown suppresses the Wnt/β-catenin signaling pathway via S1P and reprograms lipid metabolism; insufficient lipid synthesis impairs the supply of raw materials required for DNA replication during S-phase, thereby exacerbating S-phase arrest.

Dysregulated TF expression is a key upstream mechanism that regulates tumor-associated gene expression. As a core member of the AP-2 family, TFAP2A exhibits cancer type-specific functions, acting as an oncogenic driver in pancreatic cancer, triple-negative breast cancer, and esophageal carcinoma, while serving as a tumor suppressor in HCC. Notably, high TFAP2A expression in LUAD correlates with poor patient prognosis [15–18, 43, 44]. This study observed a dose-dependent effect of TFAP2A: within a specific expression range, TFAP2A promotes LUAD progression by transcriptionally activating SGPP2; however, excessive exogenous TFAP2A overexpression reduces SGPP2 protein expression, accompanied by suppressed LUAD cell proliferation and migration, as well as inhibition of the Wnt/β-catenin signaling pathway and lipid metabolism. This dual regulatory effect is hypothesized to be closely associated with the phenomenon through which the downstream molecule SGPP2 exerts feedback regulation on the upstream transcription factor TFAP2A. A PPI mediates the underlying mechanism linking SGPP2 and TFAP2A, facilitating TFAP2A degradation likely via the ubiquitin-proteasome pathway. This regulatory pattern is not uncommon in tumor-related signaling pathways. For instance, MDM2 can be transcriptionally activated by p53, while simultaneously binding to its transactivation domain of p53 and mediating its ubiquitination, ultimately leading to proteasomal degradation, a dual-function mechanism analogous to that described in previous studies [45, 46]. Nevertheless, the precise molecular mechanisms underlying the bidirectional regulation of TFAP2A and SGPP2 require further experimental validation and elucidation.

Aberrant activation of the Wnt/β-catenin signaling pathway is a critical driver of progression in various malignancies, including LUAD [47–49]. The core molecular component of this pathway, β-catenin, is phosphorylated by GSK3β in the cytoplasm and subsequently degraded. Upon pathway activation, GSK3β phosphorylation at Ser9 increases, leading to β-catenin accumulation and nuclear translocation, where it regulates the expression of downstream target genes, thereby promoting cell proliferation, migration and invasion [50–52]. Our experimental results show that SGPP2 cooperates with SGPP1 to elevate S1P levels, thereby activating the Wnt/β-catenin signaling pathway and ultimately driving the malignant progression of LUAD. To clarify the specificity and causality of this regulatory axis, we used XAV939, exogenous S1P and rescue assays to futher verify. This directly supports the functional integrity of SGPP2-S1P-Wnt/β-catenin regulatory axis. Moreover, related studies indicate that SPHK1/S1P upregulates β-catenin through S1P2 receptors [53], and GSK3β can phosphorylate SREBP1 [54], providing evidence that the lipid metabolism pathway and the Wnt/β-catenin signaling pathway interact and jointly modulate LUAD progression. The elucidation of this mechanism not only enriches the molecular regulatory network underlying LUAD but also provides a solid experimental and theoretical foundation for the future development of targeted therapeutic strategies against LUAD centered on the SGPP2-S1P-Wnt/β-catenin axis.

Lipid metabolic reprogramming in tumor cells is a core characteristic that meets the demands of rapid proliferation. Enhancing lipid synthesis, it provides sufficient raw materials for the construction of membrane structures, energy supply, and signal molecule generation in tumor cells [55, 56]. Functional experiments demonstrated that SGPP2 modulates lipid metabolic reprogramming in LUAD cells by regulating SKI1 expression, thereby influencing the SREBP1-FASN/SCD axis. This metabolic adaptation not only provides essential lipid substrates to support rapid tumor proliferation, but also enhances tumor cell migration and invasion by remodeling the TME [57], which further supports the association between high SGPP2 expression and lymph node metastasis. Notably, lipid metabolic reprogramming often exhibits synergistic regulatory interactions with Wnt/β-catenin pathway activation. The present study reveals that SGPP2 can concurrently modulate both key processes, suggesting that SGPP2 functions as a central regulatory node integrating lipid metabolism and the Wnt/β-catenin signaling pathway. This integration establishes a coordinated “metabolism-signaling” network that collectively drives the malignant progression of LUAD. However, the precise molecular mechanisms underlying this synergistic regulation require further in-depth investigations for comprehensive validation and elucidation.

### Research limitations and future directions

Although this study systematically elucidates the role and molecular mechanism of the “TFAP2A-SGPP2-lipid metabolism-Wnt/β-catenin” regulatory axis in LUAD, several limitations exist: (1) Clinical sample limitations: The clinical samples were derived from a single center with a relatively small sample size, which may have introduced a selection bias. Future studies should include multi-center clinical samples to further validate the prognostic value of SGPP2. (2) Unclear mechanism of SGPP2-Wnt/β-catenin interaction: The specific molecular mechanism by which SGPP2 regulates the Wnt/β-catenin signaling pathway remains unclear. The causal mechanism linking SGPP2 enzymatic activity to Wnt/β-catenin activation remains undefined, and whether S1P directly interacts with key proteins in this pathway remains to be elucidated. (3) The molecular mechanism underlying the inhibitory effect of TFAP2A overexpression on SGPP2 and the bidirectional regulation mode of SGPP2 in the reverse regulation of the upstream transcription factor TFAP2A remains unclear. Subsequent studies are needed to analyze the core components and signal feedback loops of the mutual regulation of TFAP2A and SGPP2, and to improve the overall logic of this regulatory axis.

## Conclusion

This study comprehensively investigates the role and mechanism of SGPP2 in LUAD for the first time, identifying the “TFAP2A-SGPP2-lipid metabolism-Wnt/β-catenin " regulatory axis as a core driver of LUAD progression. This discovery not only provides a novel molecular marker for LUAD prognostic assessment but also offers a potential therapeutic target for precision targeted-therapy, laying a theoretical foundation for subsequent clinical translation research.

## Supplementary Information

Below is the link to the electronic supplementary material.

Supplementary Material 1

Supplementary Material 2

Supplementary Material 3

Supplementary Material 4

## Data Availability

All data generated or analyzed during this study are included in this published article and its supplementary information files.

## Abbreviations

LUAD: Lung adenocarcinoma
TFAP2A: Transcription factor AP-2α
SGPP2 / SPP2: Sphingosine-1-phosphate phosphatase 2
SGPP1/SPP1: Sphingosine-1-phosphate phosphatase 1
UBE2T: Ubiquitin-conjugating enzyme E2 T
qRT-PCR: Quantitative real-time polymerase chain reaction
WB: Western blotting
IHC: Immunohistochemistry
CCK-8: Cell Counting Kit-8
EdU: 5-ethynyl-2′-deoxyuridine
TC: Total cholesterol
TG: Triglyceride
ChIP: Chromatin immunoprecipitation
CoIP: Co-Immunoprecipitation
EMT: Epithelial–mesenchymal transition
GSK3β: Glycogen synthase kinase 3β
NSCLC: Non-small cell lung cancer
HCC: Hepatocellular carcinoma
S1P: Sphingosine-1-phosphate
TME: Tumor microenvironment
DEGs: Differentially expressed genes
log₂FC: Log₂ fold change
GO: Gene Ontology
BP: Biological Process
CC: Cellular Component
MF: Molecular Function
KEGG: Kyoto Encyclopedia of Genes and Genomes
OS: Overall survival
RFS: Recurrence-free survival
PFI: Progression-Free Interval
GSEA: Gene Set Enrichment Analysis
TFs: Transcription factors
RT: Room temperature
HRP: Horseradish peroxidase
PVDF: Polyvinylidene fluoride
OD: Optical density
FBS: Fetal bovine serum
MUT: Mutant
WT: Wild-type
CI: Confidence interval
HR: Hazard ratio
TTF-1: Thyroid transcription factor-1
CK-7: Cytokeratin 7
p-GSK3β: Phosphorylated GSK3β (Ser9)
VEGF: Vascular endothelial growth factor
