# Supplementary figures and images for: TFAP2A regulates SGPP2 transcription to promote lipid accumulation and activate the Wnt/β-catenin signaling pathway to promote malignant progression in lung adenocarcinoma
Source: J Transl Med. 2026 Mar 4;24:490. doi: 10.1186/s12967-026-07949-x (PMC13067397; doi:10.1186/s12967-026-07949-x)

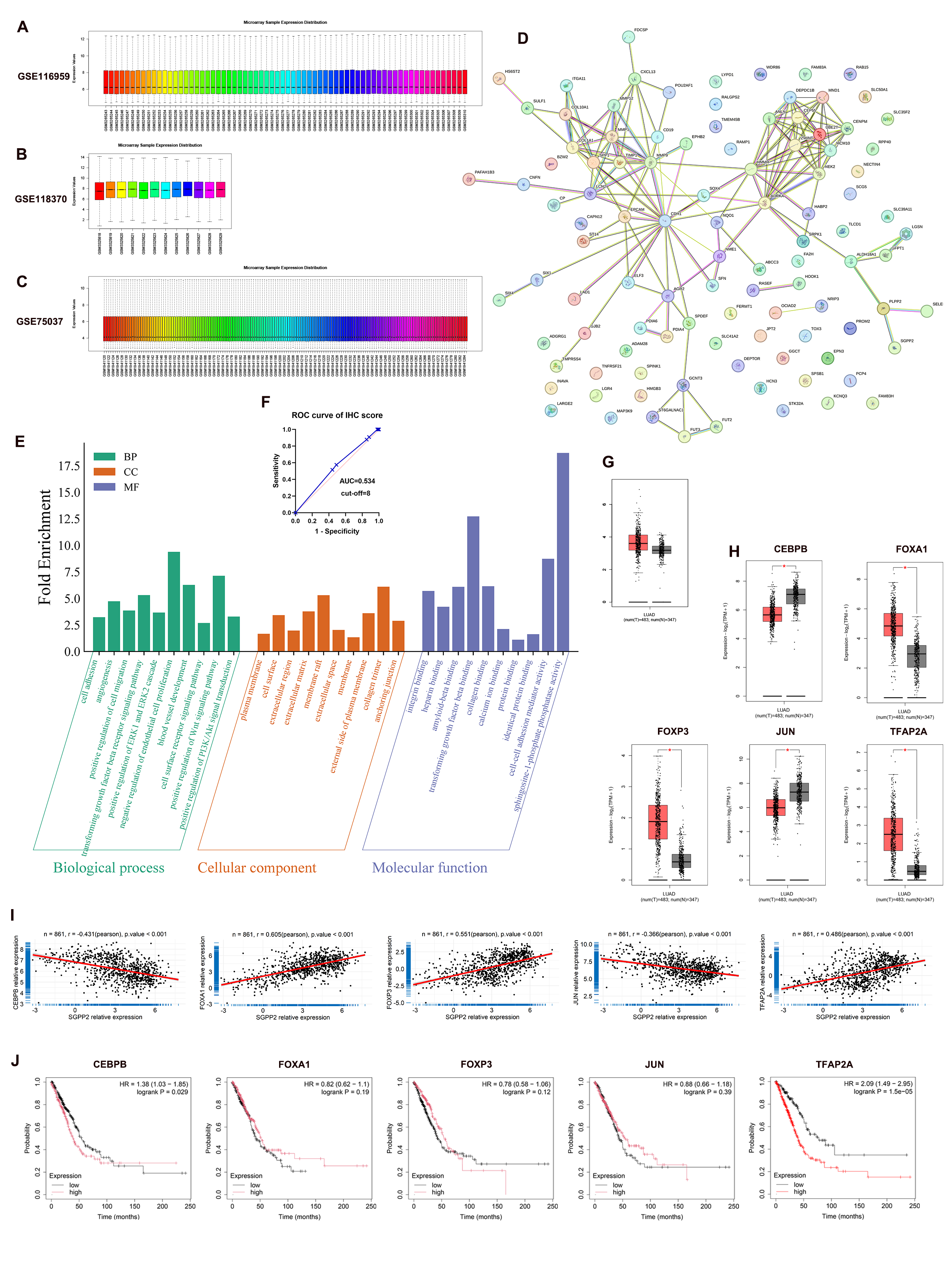

Supplement: Supplementary file 1 — Supplementary Material 1 [file 12967_2026_7949_MOESM1_ESM.tif]

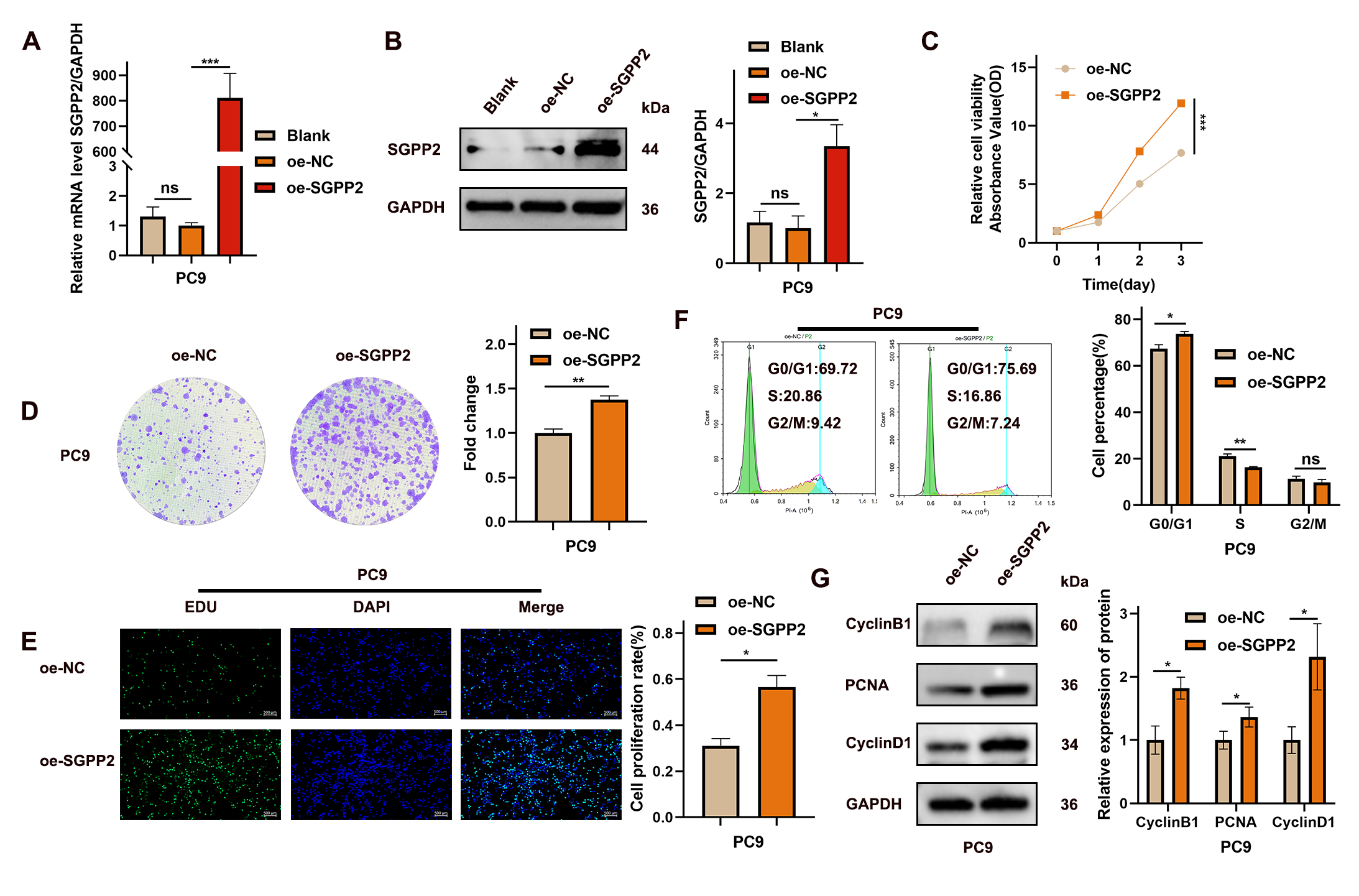

Supplement: Supplementary file 2 — Supplementary Material 2 [file 12967_2026_7949_MOESM2_ESM.tif]

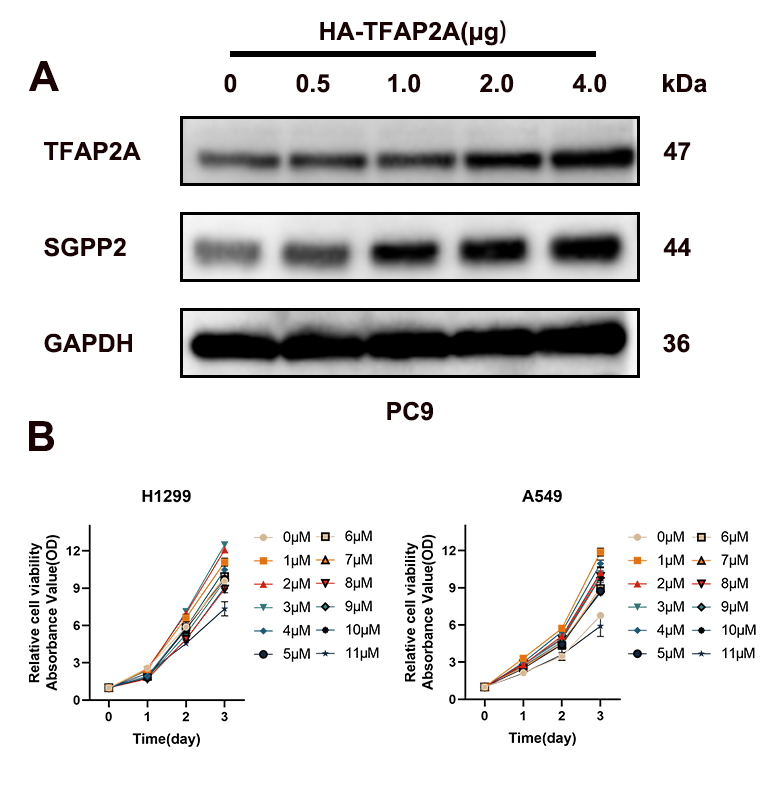

Supplement: Supplementary file 3 — Supplementary Material 3 [file 12967_2026_7949_MOESM3_ESM.tif]
