# Supplementary material for: TFAP2A regulates SGPP2 transcription to promote lipid accumulation and activate the Wnt/β-catenin signaling pathway to promote malignant progression in lung adenocarcinoma
Source: J Transl Med. 2026 Mar 4;24:490. doi: 10.1186/s12967-026-07949-x (PMC13067397; doi:10.1186/s12967-026-07949-x)
Supplement: Supplementary file 4 — Supplementary Material 4 [file 12967_2026_7949_MOESM4_ESM.docx]

**Supplementary Table 1:** siRNA and Primer Sequences

| Name | Spaices | Forward (5’-3’) | Reverse (5’-3’) |
| --- | --- | --- | --- |
| si1-TFAP2A | human | CAUCACUAGUAGAGGGAGATT | UCUCCCUCUACUAGUGAUGTT |
| si2-TFAP2A | human | CGCCAAAAGCAGUGACAAATT | UUUGUCACUGCUUUUGGCGTT |
| si-NC | human | UUCUCCGAACGUGUCACGUTT | ACGUGACACGUUCGGAGAATT |
| GAPDH | human | GGAAGCTTGTCATCAATGGAAATC | TGATGACCCTTTTGGCTCCC |
| SGPP2 | human | ATTGCCTTCACCCTCCTTATCT | CAGGAAGAATGGCACAACTATGA |
| TFAP2A | human | CAGGAAGAATGGCACAACTATGA | ATCGGAATGTTGTCGGTTGAG |
| ChIP-PCR | human | TTAACACATAAGCAGAACAGGTCC | CAGGAGGTGGAAGCATCTCT |

**Supplementary Table 2:** GSEA enrichment analysis revealed that SGPP2 might affect lipid metabolism in LUAD

| Name | NES | NOM p-val | FDR q-val |
| --- | --- | --- | --- |
| GLYCEROPHOSPHOLIPID METABOLISM | 2.096927 | 0 | 0.001489 |
| GLYCEROLIPID METABOLISM | 2.081634 | 0 | 0.002491 |
| ARACHIDONIC ACID METABOLISM | 1.674791 | 0.015686 | 0.019206 |
| FATTY ACID METABOLISM | 1.631524 | 0.037182 | 0.026748 |
| ALPHA LINOLENIC ACID METABOLISM | 1.591849 | 0.040241 | 0.034431 |

**Supplementary Fig. 1** (A–C) Microarray sample expression distribution of three datasets (GSE75037, GSE118370, and GSE116959); (D) PPI network of DEGs; (E) GO enrichment analysis of DEGs; (F) ROC curve plotting the relationship between the SGPP2 IHC score as the test variable and OS as the outcome variable; (G) Expression of SGPP1 in LUAD from the GEPIA2 database; (H) Expression of upstream TFs in LUAD (GEPIA2 database); (I) Correlation between upstream TFs and SGPP2; (J) Association between expression of upstream TFs and OS in LUAD. **⁎**p < 0.05; **⁎⁎**p < 0.01; **⁎⁎⁎**p < 0.001.

**Supplementary Fig. 2** SGPP2 overexpression promotes LUAD cell proliferation. (A, B) Validation of SGPP2 overexpression efficiency in PC9 cells (qRT-PCR and WB). (C–E) Effects of SGPP2 overexpression on cell proliferation (CCK-8 assay, colony formation assay, EdU assay). (F) Effects of SGPP2 overexpression on cell cycle distribution (flow cytometry). (G) WB analysis of cell cycle-related proteins after SGPP2 overexpression. **⁎**p < 0.05; **⁎⁎**p < 0.01; **⁎⁎⁎**p < 0.001.

**Supplementary Fig. 3** (A) The expression of SGPP2 was positively correlated with the plasmid amount of TFAP2A in the 0–4ug range; (B) CCK-8 assay was used to verify the effects of different concentrations of S1P on the proliferation of A549 cells and H1299 cells.
